# CISBETI 2019 - International Congress of Health, Well-Being, Technology and Innovation: Foz de Iguaçu, Brazil. 4-6 April 2019

**DOI:** 10.1186/s12913-019-4213-z

**Published:** 2019-07-15

## A1 Notification of adverse events in teaching hospital: Retrospective study from 2013 to 2018

### Luciene M Braga^1^, Eliza C Alves^1^, Andréia G Siman^1^, Marilane O Fani^1^, Fernanda B O Santos^2^

#### ^1^Federal University of Viçosa, Minas Gerais, Brazil; ^2^Federal University of Minas Gerais, Minas Gerais, Brazil

##### **Correspondence:** Luciene M Braga (luciene@daskaleas.com)

**Introduction:** Adverse events are incidents with the potential for temporary or permanent damage and risk of death to the patient, due to a failure in safety. Continuously monitoring the adverse events related to health care is essential for the institution to identify its weaknesses and develop actions for improving care practices, ensuring the patient safety.

**Objective:** To analyze the notifications of adverse events in a teaching hospital in Brazil between 2013 and 2018.

**Method:** a descriptive and retrospective study, carried out by the analysis of 122 notifications of adverse events between January 2013 and August 2018, in a teaching hospital in a small city of Minas Gerais-Brazil. The statistical analysis considered absolute and relative frequencies by means of SPSS 25.0.

**Results:** The professionals made note of six adverse events in 2013, 14 in 2014, 34 in 2015, 28 in 2016, 17 in 2017 and 23 until August 2018. Adverse events were reported mainly by nurses (60.7%) and related to: errors in the preparation and administration of medication (28.7%), falling from bed (24.6%), medical procedures (13.9%), health devices (11.5%), falls from one's height (10.7%), among others (10.6%). The people involved in the adverse events were the nursing assistant (68%), nurses (13.1%), the patient's companion (5.7%), the dietician (2.5%) and the doctor (1.6%), with 9% being not reported. All patients were assisted by the doctor after the occurrence of the adverse event and two events resulted in death.

**Conclusion:** Notifications almost doubled between 2014 and 2016, there being a fall in 2017, this fact reinforcing the need to sensitize professionals to carry out the notification. The adverse events occurred mainly with the members of the nursing team, involving medication errors and patient falls, which are preventable. Therefore, the nine rights of medication, the elaboration of protocols and capabilities are essential actions to be implemented to reduce the rates of adverse events.

**Keywords:** Patient Safety; Quality Indicators, Health Care; Nursing

## A2 The nursing process for inpatient units: Software adaptation

### Camila S Domingos^1^, Gabriela T Boscarol^1^, Cristiane C Souza^1^, Meire C Tannure^2^, Tânia C Chianca^3^, Bruno D Henriques^1^, Luciene M Braga^1^, Patrícia O Salgado^1^

#### ^1^Federal University of Viçosa, Minas Gerais, Brazil; ^2^Pontifical Catholic University of Minas Gerais, Minas Gerais, Brazil; ^3^Federal University of Minas Gerais, Minas Gerais, Brazil

##### **Correspondence:** Camila S Domingos (camilasantanadomingos@gmail.com)

**Introduction:** The Resolution 358/09 of the Federal Nursing Council-Brazil recommends that nursing care be systematised, grounded in a theoretical support that guides the steps of the nursing process [1]. The use of software has been indicated for the operationalization of the nursing process. Thus, a software called Information System with the Nursing Process in Intensive Therapy (SIPETi) based on Basic Human Needs Theory has been developed [2].

**Objective:** To adapt the data collection stage of the SIPETi software for use in unit of a medical-surgical clinic.

**Method:** Descriptive study developed in a unit of a medical-surgical clinic of a philanthropic hospital in Minas Gerais, Brazil. Collection of data was carried out between January and March 2017, the intentional sample comprised 100 patients. Methodology involved three stages. The first was to empower researchers through inter-observer training. In the second stage the software was employed on the first day of admission, followed by daily monitoring of the patient until discharge, transfer or death. 57 patients were registered at the female clinic and 43 in the male one. The third step consisted in the analysis of the software, screen to screen, to identify the necessary modifications. Data were analyzed through descriptive statistics.

**Results:** In the module of patient registration the exclusion of one (5%) item was suggested, six changes were included (30%) and four changes were proposed (20%). In the four screens that comprise the anamnesis, no item was deleted; 26 (48.14%) inclusions and seven (12.96%) changes were proposed. In the ten screens of the physical examination the exclusion of 31 items (22.46%), the inclusion of 26 (18.84%) and 27 changes were suggested (19.56%).

**Conclusion:** The study allowed for the adaptation and improvement of the SIPETi software to record the first step of the nursing process of patients admitted to a medical-surgical clinic.

**Keywords:** Nursing; Nursing Process; Nursing Informatics; Software Validation; Software.

**References**

1.Cofen - Conselho Federal de Enfermagem. Resolução n° 358/2009, de 15 de outubro de 2009.

2. Martins MCT, Chianca T. Construction of a software with the nursing process in intensive care. J Health Inform. 2016 Oct-Dez; 8(4):119-25.

## A4 Incidence of unstable glycemia in critically ill patients

### Lídia M Brinati^1^, Carla F Januário^2^, Paula C Balbino^2^, Tiago R Moreira^2^, Sílvia A Cardoso^2^, Luciene M Braga^2^, Patrícia O Salgado^2^

#### ^1^São Sebastião Hospital, Viçosa, Minas Gerais, Brazil; ^2^Federal University of Viçosa, Minas Gerais, Brazil

##### **Correspondence:** Lídia M Brinati (lmbrinati@hotmail.com)

**Introduction:** Unstable glycemia is a nursing problem that deserves attention in clinical practice in intensive care units (ICU). The nursing diagnosis risk of unstable glycemia is defined by NANDA International, as the “susceptibility to variation of glucose serum levels in relation to the normal range, which can compromise health” [1]. In critical patients, the goals of glycemic levels vary from 140 to 180 mg/dL, preventing blood glucose levels below 100 mg/dL [2].

**Objective:** To estimate the incidence of unstable glycemia in adult patients admitted to an ICU.

**Method:** Cohort study conducted in an adult ICU between March and July 2017. A sample of 62 patients who were monitored daily by a nurse, since their admission to release, death or transfer. Clinical and therapeutic was followed and a sample of venous blood for measurement of blood glucose was collected. Biochemical analysis was performed on the blood samples. Patients with unstable blood glucose levels were evaluated and prescribed therapy as per institutional protocol. Descriptive analysis of the data in SPSS 23.0 was performed and the incidence of unstable blood glucose, hypoglycemia and hyperglycemia was determined.

**Results:** Among the 62 patients who participated in the study, 31 were females and the majority (64.52%) were older than 60. A predominance of patients with medical diagnosis of diseases of the circulatory system was found (32.26%). The average value of hypoglycemia was 58.9 mg/dL and hyperglycemia was 217.5 mg/dL. 28 patients had unstable glycemia, the incidence rate was 45.16%, with 22.58% having hypoglycemia and 22.58% hyperglycemia.

**Conclusion**: The incidence of unstable blood glucose in patients hospitalized in the ICU adults was high, occuring in nearly half of the patients. This result reinforces the need to know the risk factors so that nursing care specific to the clinical condition of the patients for the prevention of this problem is implemented.

**Keywords:** Hyperglycemia; Hypoglycemia; Critical Care; Nursing Diagnosis; Nursing.

**References**

1. Herdman TH, Kamitsuru S, Nanda International. NANDA International Nursing Diagnoses: Definitions and Classification 2018-2020. New York: Thieme. 2018.

2. Sociedade Brasileira de Diabetes (SDB). Controle da hiperglicemia intra-hospitalar em pacientes críticos e não críticos. Cap. 10. [cited Oct 27, 2018].

## A5 The care of hypertensive patients in primary care in a small city of Minas Gerais-Brazil

### Fernanda B O Santos^1^, Tamires A Santos^2^, Fernanda A S Carregal^3^, Andréia G Siman^4^, Luciene M Braga^4^

#### ^1^Federal University of Minas Gerais, Minas Gerais, Brazil; ^2^College of Sete Lagoas –UNIFEMM, Minas Gerais, Brazil; ^3^College of Minas –FAMINAS BH, Minas Gerais, Brazil; ^4^Federal University of Viçosa, Minas Gerais, Brazil

##### **Correspondence:** Fernanda B Santos (fernandabosufmg@gmail.com)

**Introduction:** Hypertension is a major public health problem in Brazil. Hypertensive patients need to have their blood pressure values accompanied to control this chronic ailment. The experience in clinical teaching by nursing academics has been pointing out to a weakness in the measurement of blood pressure by nurses and nursing technicians.

**Objective:** To analyze the perception of nurses in relation to their work and their team in the intervention, prevention and promotion of health for hypertensive patients.

**Method:** The data were collected in the second half of 2017, in five basic health units in a small city of Minas Gerais, Brazil, which had health education activities geared to hypertensive patients. Structured interviews with nurses and a field diary were conducted, followed by content analysis [1].

**Results:** Three categories emerged: “The (lack of) knowledge of technique”, “The time of the professional” and “Attention not integral to hypertensive patients”. The participants affirm that their teams know the technique recommended to evaluate blood pressure, however they recognize not performing it fullydue to lack of time. Integral care, according to the participants, is also impaired due to lack of time and lack of governability for access to complementary exams. However, health education activities focused on lectures that alienate the prescription of anti-hypertensive drugs to the users’ participation in these activities.

**Conclusions:** Incorrect measurement of arterial pressure and not integral approach of hypertensive patients by primary care nursing may have serious consequences in the aggravation of the disease, since it is a relevant procedure for the prevention of cardiovascular and neurological diseases. The nurses and their team lose the opportunity to perform comprehensive approaches to patients, with the creation of effective links to the health service that could contribute to the goal of integral care that is beyond requesting supplementary examinations.

**Keywords:** Hypertension; Nursing Care; Integrality in Health.

**Reference**

1. Bardin L. Análise de conteúdo. 3ª reimp. Lisboa: Edições; 2011.

## A6 Nursing care to elderly victims of ischemic stroke in an emergency care unit in a small city of Minas Gerais-Brazil

### Fernanda B O Santos^1^, Ana C Pereira^2^, Fernanda A S Carregal^3^, Andréia G Siman^4^, Luciene M Braga^4^

#### ^1^Federal University of Minas Gerais, Minas Gerais-Brazil; ^2^University Center of Sete Lagoas (UNIFEMM), Minas Gerais-Brazil; ^3^Minas Faculty (FAMINAS), Minas Gerais-Brazil; ^4^Federal University of Viçosa, Minas Gerais-Brazil

##### **Correspondence:** Fernanda B Santos (fernandabosufmg@gmail.com)

**Introduction:** Considering that ischemic stroke is one of the main issues affecting the elderly in Brazil and in the world, and that the nurse is an important agent for preventing major health grievances of these subjects, the present study was undertaken.

**Objective:** To analyze the care provided by nurses to the elderly victim of ischemic stroke in the perspective of the nursing professional in a hospital emergency care in the small city of Minas Gerais-Brazil.

**Method:** Descriptive, qualitative research, semi-structured interviews with eight nurses who work in an emergency care service in a small city of Minas Gerais-Brazil. The findings were treated according to content analysis [1].

**Results:** Three categories emerged: “Recurring care proposed by nurses”; “Risks and complications of ischemic stroke during hospitalization”; “Continuity of care for the elderly victim of ischemic stroke after hospital discharge”. In general, hospitalizations for strokes are long and the basic care have been neglected by the nurse, compromising integral assistance. Despite some assertive propositions for the care of these patients, nurses and his team do not perform them as frequently as needed. They attribute this situation of neglect of care to the lack of time, work overload and lack of human resources. As it is common for the patient in these conditions to remain in a weakened state, and may have multiple sequels, the continuity of care for the elderly should be guaranteed by means of a plan which will serve as a reference for initial care which should be referenced to primary care.

**Conclusion:** Nursing care for the elderly victim of vascular accidents is not systematised, there being negligence in care. The rationale for the negligence involves insufficient human resources, leading to work overload and lack of time to provide the care integrally. Systematizing assistance can support the pursuit of more favorable prognoses for integral assistance to the elderly victim of ischemic stroke.

**Keywords:** Brain Ischemia; Stroke; Nursing; Comprehensive Health Care.

**Reference**

1. Bardin L. Análise de conteúdo. 3ª reimp. Lisboa: Edições; 2011.

## A7 Academic league of community health: Strengthening of the triad of university teaching, research and extension

### Fernanda A S Carregal^1^, Fernanda B O Santos^2^, Thiago F Diniz^2^, Andréia G Siman^3^, Luciene M Braga^3^

#### ^1^Minas Faculty (FAMINAS), Minas Gerais-Brazil; ^2^Federal University of Minas Gerais, Minas Gerais-Brazil; ^3^Federal University of Viçosa, Minas Gerais-Brazil

##### **Correspondence:** Fernanda A Carregal (fernanda.carregal@hotmail.com)

**Introduction:** Academic Leagues are student organizations that, under the guidance of professors, carry out activities of teaching, research and university extension in a specific area of health, expanding the action horizons. In this context, the Academic League Of Community Health (LASC) is notable as a powerful tool for collective growth, favoring the emergence of a critical reflective point of view and the training of future competent professionals, committed to care integrality. It has a multidisciplinary team composed by academics of the nursing, biomedicine and medicine courses.

**Objective:** Report on the activities carried out by the academic league of community health and its relationship with the university triad.

**Method:** Experience reports. The report of the activities required by academics from the Coordination of Extension of the teaching institution to which the league is bound, between November 2016 and October 2018 was used.

**Results:** It was observed that the activities carried out in the academic league allowed its members to deal with issues related to community health (teaching), participate in projects of scientific initiation (research) and promote actions outside the Community (extension). In this way, the acquisition of knowledge related to education in health was given, and skills in the area were developed. After an analysis of the records of the activities of the academic league, the progress concerning scientific production was noted: 2016 with five abstracts published in events annals (15.1%), 2017 with sixteen (48.5%) and 2018 with twelve (36.4%). The evolution of academic production is a result of the implementation of research meetings that encourage the spread of the participants’ scientific knowledge. In addition, there was implementation of extending actions in a multidisciplinary team with an emphasis in situations that afflict the community.

**Conclusions:** The insertion of the academic league offers benefits to both the training of students as well as to the local and regional population in which it is inserted, favoring the consolidation of the university triad. Through its activities, the LASC provides the deepening of knowledge related to community health, besides enabling the development of key competences in health among the participating students.

**Keywords:** Professional Training; Health Promotion; Education.

## A8 Philosophical-conceptual structure basing the approach of people in hemodialysis according to Artinian: Implications for the care

### Jéssica C Santos^1^, Cristina Arreguy-Senna^1^, Carla A Cruz^1^, Pâmella M Reis^1^, Luciene M Braga^2^, Fernanda Krepker^1^, Marcos G Brandão^3^

#### ^1^Federal University of Juiz de Fora, Minas Gerais-Brazil; ^2^Federal University of Viçosa, Minas Gerais-Brazil; ^3^Federal University of Rio de Janeiro, Minas Gerais-Brazil

##### **Correspondence:** Jéssica C Santos (jessicacastroenf@gmail.com)

**Introduction:** Hemodialysis is a renal substitution therapy procedure that in Brazil is predominantly performed by people from 19 to 64 years of age and male, impacting on personal, productive, social and family life, which motivates a guided approach to minimize its impact.

**Objective:** To reflect on the concepts, assumptions and implications of the Artinian intersystem referential applied to the structuring of the nursing consultation with people on hemodialysis.

**Method:** Theoretical study, based on concept analysis, synthesis of assertion and derivation of the applicability of the theoretical references in the clinical practice of nursing with people on hemodialysis performed through a workshop using the technique of information translations executed in Oct/Nov.2018 with undergraduate and graduate nursing students in the course titled: “Philosophical, conceptual and theoretical bases for nursing”. It was theoretical reference adopted to Artinian Theory.

**Results:** An instrument built aiming to guide the data collection in people in hemodialysis, and by the cross-mapping technique were construct the following diagnoses, interventions and results making use of NANDA-International, NIC and NOC taxonomies, all of them structured according to the Artinian model. In order to select the content to be approached was used the one mentioned in Arreguy-Sena et al. [1]; which allowed to translate the nursing care to individuals in hemodialysis.

**Conclusions:** The concepts and proposed assumptions by Artinian when applied to nursing consultation structure proved to be feasible for the approach of an individual in hemodialysis, allowing graduate/postgraduate students to direct and give intentionality to the care for people on hemodialysis, and grounding the nursing care planning in a conceptual and philosophical theoretical framework of the nursing area. The approximation of the same with the explanatory models of nursing practice constitutes a technology compatible with the nurses’ performance.

**Reference**

1. Arreguy-Sena, C et al. Construção e validação de impressos: sistematização do cuidado de pessoas em hemodiálise. *Rev. Bras. Enferm.* [online]. 2018, 71(2):379-390.

## A9 Vulnerability self-perception for fall at home: Implications for the health first care team

### Paulo Pinto^1^, Jéssica C Santos^1^, Marcos G Brandão^2^, Pedro M Parreira^3^, Luciene M Braga^4^, Cristina Arreguy-Sena^1^, Elenir P Pereira^1^

#### ^1^Federal University of Juiz de Fora, Minas Gerais, Brazil; ^2^Federal University of Rio Janeiro, Minas Gerais, Brazil; ^3^Health Sciences Research Unit: Nursing (UICISA:E) Nursing School of Coimbra, Coimbra, Portugal; ^4^Federal University of Viçosa, Minas Gerais, Brazil

##### **Correspondence:** Paulo Pinto (paulo.ferpinto@gmail.com)

**Introduction:** The fact that the falls are multifactorial events and present in the home environment motivated the need to identify the situations considered stressful by the participants, those being mentioned or observed by the health professional.

**Objective:** To analyze intrapersonal, interpersonal and extrapersonal stressors of falling at home according to Neuman’s system theory and to elaborate a protocol from a situational diagnosis to subsidize the approach of professionals of the health team in the prevention of falls among elderly people.

**Method:** Participatory observation concomitant with a sectional study with a full sample selection performed at home. Inclusion criteria were: individuals aged ≥65 years, assigned to a Primary Health Care Unit. Data collection instrument: sociodemographic characterization, household, illness, visual capacity and vulnerability for falls profiles. Data collected by individual interviews with support of the Open Date Kit and analyzed with the support of SPSS® software (descriptive and correlational statistics). Met ethical and legal requirements.

**Results:** 220 elderly people participated in this study: 78.5% women; 29.6% with age ≥80 years (65-96 years). Snellen's scale showed visual impairment in 54.1% (left eye: LE) and 50.6% (right eye: RE) with reading ability ≤5 or 20/40 diopter; Jaeger's scale measured that 55.2% and 53.2% participants did not see anything or saw poorly LE and RE; 41.6% perceived poorly with LE and RE. Falls Efficacy Scale 53.5% evidenced fear for activities of daily life, being a predictor of falls. The following environmental factors were involved in the fall: at home (slippery floor, ladder and handrail with p-value ≤ 0.04) and outside home (many objects in the environment and steps with p-value ≤ 0.04). Participatory observation enabled the professional to identify and analyze the perception of the elderly in the situations/circumstances of vulnerability.

**Conclusions:** The present investigation outlined a region covered by Primary Health Care on vulnerabilities for falls in people aged ≥65 years, presented a protocol (soft-hard technology) to subsidize multi-professional approach and management decisions on care for the outside of home with a prevention approach for the occurrence of falls.

**Keywords:** aged, fall, health vulnerability

## A10 Nursing consultation with people in pre-renal transplantation technology based on modeling/role-modeling

### Cristina Arreguy-Sena^1^, Romanda B Lemos^1^, Thaís O Santos^1^, Nathalia O Martins^1^, Micheli F Cruz^2^, Fernanda K Ferreira^1^, Tainá Oliveira^1^

#### ^1^Federal University of Juiz de Fora, Minas Gerais, Brazil; ^2^Nursing School Anna Nery, Minas Gerais, Brazil

##### **Correspondence:** Cristina Arreguy-Sena (cristina.arreguy@ufjf.edu.br)

**Introduction:** The difficulty of people in renal transplantation to maintain adherence to therapeutic recommendations may be influenced by the distortion of understanding and false expectations of which are the demands that will arise after transplantation, being the nursing consultation a technological and operational strategy that can contribute to overcome this gap in nursing care.

**Objective**: To describe the construct of a technological strategy to approach people in the pre-renal transplant phase in the nursing consultation modality.

**Method:** Theoretical study, based on the desire to respond to the results of the analysis of the need to include guidelines for transplanted people in the technological modality of the Nursing Consultation, using interactionist processes. The following criteria were used: content capable of modeling knowledge, conduct and behaviors of people transplanted during the pre-transplantation period in order to maximize their adherence and resilience to the renal transplantation process. Activities developed in Oct/Nov 2018 in the course “Philosophical, conceptual and theoretical bases for nursing”, when a workshop was performed with application of the techniques: information translations and cross-mapping with the participation of undergraduate and graduate students in nursing were carried out. Theoretical references were: Theory of Erickson, Tomlin and Swain. Due to the profile of the study there was no need to submit it to the Ethics Committee.

**Results:** From the analysis of the needs of inclusion of guidelines to the transplanted people, using the technology of the Nursing Consultation according to interactionist processes, it was possible to elaborate three instruments (data collection, possible nursing diagnoses and interventions and nursing results) based on the Modeling/Role-Modeling proposed by Erickson, Tomlin and Swain and in the North American Nursing Diagnosis Association, Nursing Intervention Classification and Nursing Outcome Classification respectively. By cross-mapping it was possible to connect 11 diagnoses, interventions and nursing results.

**Conclusions:** The constructed instruments (data collection, possible diagnoses, interventions and nursing results) are compatible with the approach of renal transplant candidates and aim to provide them with an enlightening approach in a welcoming environment that favors the resilience to the transplantation process and constitute an opportunity to clarify the conditions and lifestyle they will face after transplantation.

**Keywords**: Kidney Transplantation, Office Nursing, Nursing Theory

## A11 Peripheral venipuncture in hospitalized individuals: Gibi technique supporting processual approach of social representations

### Fernando C Ribeiro^1^, Cristina Arreguy-Sena^1^, Antônio T Gomes^2^, Luciene M Braga^3^, Paula Krempser^1^, Laércio D Melo^1^, Michele N Melo^1^

#### ^1^Federal University of Juiz de Fora, Minas Gerais, Brazil; ^2^Estadual University of Rio Janeiro, Minas Gerais, Brazil; ^3^Federal University of Viçosa, Minas Gerais, Brazil

##### **Correspondence:** Fernando C Ribeiro (fernandoenfer@gmail.com)

**Introduction**: During the period of hospitalization, veins are often punctured for therapeutic purposes, and the sharing of the perceptions of those hospitalized about this procedure is a valuable component for the structuring of nursing care, since they portray the human responses of a socially constituted group.

**Objective:** To understand the symbolic constructs about peripheral venipuncture by hospitalized individuals who have had their veins punctured.

**Method:** Research structured in the procedural approach of the Theory of Social Representations according to Moscovici performed at a private hospital with Unified Health System beds. Participants were hospitalized people in the clinical and surgical sectors, aged ≥ 18 years who had their veins punctured for therapeutic purposes. An interview with audio recording supported by the technique of clipping and collage of comic strips triggered by guiding question (How is it to have the vein punctured for treatment). Provided 12 sheets of plasticized comic material to choose a figure from. Discourse contents treated by content analysis according to Bardin with support from the NVivo Pro11® Software. Ethical aspects of research were met.

**Results:** 117 hospitalized individuals participated. The comic figures selected by the participants motivated approximations with the theme, with six thematic categories identified: (1- the intravenous catheter, the discomfort and the local evaluation; 2- the treatment and recovery; 3- the needle, the expectation and the puncture; 4- the reason for the puncture and personal reactions; 5- the relationship with professionals and 6- the punishment, the illness-death and religious devotion). These content emerged from personal experience, with hospitalized people and in contact with professionals and were illustrated with selected comic figures and speech fragments that exemplified them.

**Conclusions:** The symbolic constructs on peripheral venipuncture made it possible to identify positive, neutral and negative reactions, the latter being the focus of reflections of nurses when planning nursing care, or the nursing team during venipuncture, maintaining, evaluating, manipulating, and removing intravascular catheter.

**Keywords:** Vein puncture Nursing Care, Hospitalization, Nursing Theory

## A12 Knowledge and information of users about imaging tests with contrast: Pre-examination assistance

### Romanda B Lemos^1^, Cristina Arreguy-Sena^1^, Luciene M Braga^2^, Anna P Pereira^1^, Amanda M Marangon^1^, Letícia L Pereira^1^, Thainá R Morais^1^

#### ^1^Federal University of Juiz de Fora, Minas Gerais, Brazil; ^2^Federal University of Viçosa, Minas Gerais, Brazil

##### **Correspondence:** Romanda B Lemos (romanda.barboza@gmail.com)

**Introduction**: Computed tomography and magnetic resonance imaging tests when the images are inconclusive require the use of contrast, and diagnostic imaging services are a specialized sector in which the insertion of the nurse can contribute to the reception and adaptation of the users to the recommended service in these sectors.

**Objective:** To understand the information and knowledge of people who are users of a diagnostic imaging sector under tests of computed tomography (CT) and magnetic resonance imaging (MRI), with contrast.

**Method:** Descriptive study with a qualitative approach conducted in a diagnostic imaging center. Participants were users of the Unified Health System submitted to contrast-enhanced computed tomography or magnetic resonance imaging tests. The data collection instrument was structured by sociodemographic characterization, audio recording interview prompted by guided questions: How is it for you to be here and be examined in a test with contrast? Data consolidation in Programa NVivo Pro11® software and analyzed according to Bardin content analysis. There were theoretical deepening by Pearson correlation ≥0,7. Were met all ethical/legal research requirements involving human beings. (protocol no 2.633.992).

**Results:** Participated 33 people submitted to computed tomography and magnetic resonance imaging. From speech analysis surfaced two categories: 1) needle insertion and contrast 2) the exam and its results. The use of contrast concern a few participants, being the pre-exam guidance being condition that reassure them since they can perceive what will happen to them which favors their coping with the situation. The possibility of an extravasation is seen as an occasional event and to take the test can be of much less concern compared to the uncertainties of the test results.

**Conclusions:** the information and knowledge of people submitted to CT and MRI reveal concerns that are able to be dealt with during the welcoming and pre-examination phase, which is compatible with the nurse practice in this environment. We suggest the structuring of the assistance system to guide the professional approach.

**Keywords:** Diagnostic Imaging, Contrast Media, User Embracement, knowledge

## A13 Approach of people who smoke in the light of Artinian: Implications for the nursing consultation

### Cássia E Delgado, Cristina Arreguy-Sena, Romanda B Lemos, Carolina O Baumgratz, Anna B James, Anna P Nogueira, Beatrys R Menezes

#### Federal University of Juiz de Fora, Minas Gerais, Brazil

##### **Correspondence:** Cássia E Delgado (cassia_evan_delgado@hotmail.com)

**Introduction:** The structuring of the nursing care by the means of epistemological constructs consists in a need and allows the nurse to translate the aiming and intentionality they apply to their therapeutic actions, the theoretical models serving as explanatory forms to base their practices.

**Objective:** To reflect on the conceptual approach, assumptions and implications of the Artinian Intersystem Model when applied to structure the nursing consultation with active/passive smokers assigned to Primary and Secondary Health Care Units (Brazil).

**Method:** Theoretical study, based in concept analysis, assertion and derivation of the applicability of the theoretical references in the clinical practice of nursing with active/passive smokers performed through an workshop using the translation of information technique performed in Oct/Nov.2018 with undergraduate and graduate nursing students in the elective course entitled: “Philosophical, conceptual and theoretical basis for nursing”. The Intersystem Model of Artinian was the theoretical reference.

**Results:** Instruments were built for data sample: diagnosis (x), interventions (x) and nursing results (x), using NANDA-International taxonomy, *Nursing Intervention Classification* e *Nursing Outcome Classification* applying the concepts of the Artinian theory to base the nursing consultation. The adopted theoretical concepts, Foram conteúdos teóricos adotados, the ones recommended by the Ministry of Health and by INCA-Brazil, portraying a conception of nursing care in the fight against smoking.

**Conclusions:** The Artinian reference resulted compatible with the approach of structuring the nursing consultation with people aiming to quit tobacco, allowing undergraduate/graduate students to cast different views on the basis where the nursing care is structured and to get closer to explanatory models applicable to the nursing practice, as a compatible technology with the nursing acting.

**Keywords:** Tobacco Use Disorder, Nursing Theory, Office Nursing

## A14 People in medical imaging: Instrument to base the nursing consultation according to Artinian

### Romanda B Lemos^1^, Cristina Arreguy-Sena^1^, Bruna M Oliveira^1^, Evilaine C Fernandes^1^, Ana C Pinto^1^, Luciene M Braga^2^, Flávia Silva^2^

#### ^1^Federal University of Juiz de Fora, Minas Gerais, Brazil; ^2^Federal University of Viçosa, Minas Gerais, Brazil

##### **Correspondence:** Romanda B Lemos (romanda.barboza@gmail.com)

**Introduction**: Technological improvements in medical imaging diagnosis justify the existence of services in which the participation of the nurse includes: user’s safety, exams preparation, the support to face the exam, the evaluation during the exam, the screening process and care in case of adverse/undesirable effects, being the nursing consultation a scientific methodology to structure professional practice.

**Objective:** To construct instruments for data collection, diagnosis, interventions, a evaluations using the Artinian reference in a model applicable to the nursing consultation.

**Method:** Theoretical study of technological idealization to base on the nursing consultation with people in diagnosis exams of medical imaging, structuring the assistance systematization. Activities performed in Oct/Nov.2018 in the course “Philosophical, conceptual and theoretical basis for nursing”, when it was done a workshop using the techniques of: translation of information and cross-mapping. Undergraduate and graduate nursing students participated, using the Artinian reference. Because it was an theoretical study it was not needed to submit it to the Ethical Committee.

**Results:** The instrument was structured in three axes (Biological, Psychosocial and Spiritual) portraying the dimensions in which the relationship between nurse-users is established. It allows the nurse to access the user’s intersystem; to acknowledge the information and doubts regarding the test, preparation, during, and post-exam care; 2) To understand which are the main concerns about the procedure; 3) to manage the performing of the exam, with or without contrast and 4) to identify the motivation to take the exam and used strategies. These information assist the nurse in accessing their intersystem (knowledge, values and behaviors), seeking alternatives to an agreed, with consensus care. Through cross-mapping were identified diagnoses, interventions and nursing results using NANDA International, NIC and NOC taxonomies.

**Conclusions:** The built instrument guides the nursing approach through an intersystem view and favours the agreement of therapeutic interventions, making evident the parameters and indicators to assess the given care. It is compatible with its use in an computerized system, being able to give direction to a nursing approach in the context of Diagnostic Imaging Services.

## A15 Levine’s and Roy’s concepts and implications to approaches of anti-tobacco nursing consultation

### Jéssica C Santos, Cristina Arreguy-Sena, Romanda B Lemos, Lara A Gomes, Laura B Benício, Antônio I Silva, Marcos A. Brandão

#### Federal University of Juiz de Fora, Minas Gerais, Brazil

##### **Correspondence:** Jéssica C Santos (jessicacastroenf@gmail.com)

**Introduction:** Smoking is a public health problem. Considering the great nursing theories when applied to clinical practice, it translates into world visions and gives visibility to the adopted metaparadigms (conception of the human being, professional performance, health-disease process and scenario in which this practice develops) constituting in a soft and soft-to-hard technology applicable to the nursing consultation.

**Objective:** To reflect on the conceptual approximations, assumptions and implications of the theoretical references of Levine and Roy applied to the structuring of the nursing consultation with active / passive smokers assigned to Primary and Secondary Health Care Units (Brazil).

**Method:** Theoretical study, based on concept analysis, synthesis of affirmation and derivation of the applicability of the theoretical references in the clinical practice of nursing with active / passive smokers performed through a workshop using the technique of information translations in Oct/Nov.2018 with undergraduate and postgraduate nursing students in the elective course entitled: “Philosophical, conceptual and theoretical bases for nursing”. Theoretical references: Levine's principles of energy conservation and Roy's Adaptation Model.

**Results:** Two data collection instruments were constructed with their respective diagnoses, interventions and results (NANDA-International, NIC and NOC taxonomies) structured in the Levine’s energy conservation principles and in the Roy’s adaptation model, maintaining the same theoretical content proposed by the Ministry of Health and INCA-Brazil allocated according to the respective theoretical references; which allowed the translation of different nursing care focus to the tobacco control approach.

**Conclusions:** Conceptual points, assumptions and applicability of the theoretical references of Levine and Roy have been identified as being feasible for structuring the nursing consultation. This experience allowed undergraduate/graduate students to cast distinctive views on the foundations that structure nursing care. Their approximation with the explanatory models of nursing practice constitutes a technology compatible with the nurses’ performance.

## A16 Health professionals’ adherence to disposable tourniquets in the peripheral venous catheterization: An action-research study in infection prevention and control

### Anabela Salgueiro-Oliveira^1^, Paulo Costa^1^, Vânia Oliveira^2^, João Graveto^1^, Nádia Osório^3^, Fernando Gama^4^, Pedro Parreira^1^

#### ^1^Health Sciences Research Unit: Nursing (UICISA:E) Nursing School of Coimbra, Coimbra, Portugal; ^2^School of Health Technology of Coimbra, Coimbra, Portugal; ^3^Biomedical Science Department of ESTeSC, Coimbra, Portugal; ^4^Centro Hospitalar e Universitário de Coimbra, Coimbra, Portugal

##### **Correspondence:** Anabela Salgueiro-Oliveira (anabela@esenfc.pt)

**Introduction:** Peripheral venous catheterization is the most frequently performed invasive procedure in clinical settings; however, it may facilitate the occurrence of infections. During the insertion of the peripheral venous catheter (PVC), health professionals resort to the use of a tourniquet, which is often reused between patients in several settings, emerging as an important reservoir for microorganisms. Therefore, this study aims to involve nurses in the adherence to disposable tourniquets use during peripheral venous catheterization.

**Methods:** Following an action-research approach, this study takes place in a Cardiology ward of a Central Hospital in Portugal. In a first phase, a scoping review on the contamination of tourniquets was undertaken. A prospective observational study was implemented on 10/04/2018, with data collection on nurses’ practices during PVC insertion and monitoring of related complications. Simultaneously, microbiological specimens are collected from the used tourniquet, insertion site and PVC tip (on removal). In the second phase, based on the results obtained, a team reflexion on current practices will be carried out. Lastly, the study will be repeated to evaluate the impact of the change in nurses’ practices on PVC contamination.

**Results**: Twenty studies were included in the scoping review undertaken, evidencing tourniquet contamination rates between 10 and 100%. The Staphylococcus was the most prevalent genus, although a notable microbiological diversity was equally evidenced, *Enterococcus* spp. and Gram negative *bacilli* such as *Klebsiella*, *Pseudomonas*, *Escherichia coli* and *Acinetobacter baumannii*. Regarding antimicrobial susceptibility, methicillin was the most widespread resistance found. Similarly, the ongoing microbiological assessment of tourniquets used in the Cardiology ward evidences an outstanding microbiological diversity. This may be explained by the current use of non-disposable tourniquets by nurses and lack of disinfection protocols between patients.

**Conclusions**: We anticipate that the results of the scoping review and of the prospective observational study may motivate nurses to integrate the use of disposable tourniquets in clinical practice, leading to lower PVC contamination rates and an improvement in patient safety.

**Keywords:** Tourniquet; Healthcare-associated infection; Nurses.

## A18 Evaluation of Mental Health First AID Training program in ungraduated nursing students

### Luís Loureiro (luisloureiro@esenfc.pt)

#### Health Sciences Research Unit: Nursing (UICISA:E) Nursing School of Coimbra, Coimbra, Portugal

**Background:** The Mental Health First Aid Educational Program (PSSM) has the potential to increase mental health literacy, reduce personal stigma, and empower individuals for their mental health.

**Objective:** To evaluate the impact of MHFA training program in Ungraduated Nursing Students

**Method:** pre-experimental study, with single group pre and post design, applied to a sample of 46 undergraduate nursing students. The Mental Health Literacy Questionnaire was applied. The data were analyzed using the software IBM SPSS (V. 24) and G-Power (V.3.1).

**Results:** At the end of the program, students showed an improvement in all components of mental health literacy about anxiety, increasing their confidence in providing first help.

**Conclusion:** The program proves to be an adequate tool for increasing the mental health literacy of nursing students.

**Keywords:** mental health literacy; anxiety; young; nursing; first aid.

## A19 Mental Health First Aid program: A pilot study in Portuguese newly graduate nurses

### Luís Loureiro^1^, Catarina Sousa^2^

#### ^1^Health Sciences Research Unit: Nursing (UICISA:E) Nursing School of Coimbra, Coimbra, Portugal; ^2^University Hospital of Coimbra, Coimbra, Portugal

##### **Correspondence:** Luís Loureiro (luisloureiro@esenfc.pt)

**Background:**

Mental Health First Aid (MHFA) is an evidence-based intervention designed to improve mental health literacy to assist to someone with a mental health problem. This pilot study, with a *one*-*group posttest* pre-experimental *design*, aims to evaluate the MHFA course in newly graduates in nursing.

**Methods**

The sample was constituted by 16 newly Portuguese graduates in nursing, with an average age of 21,86 years and standard deviation=0,54 years, randomly selected and submitted to Mental Health First Aid during two days (15 hours) in August of 2014.

To assess the mental health literacy related to MHFA program, mental health first aid intentions and confidence to help someone experiencing a mental health problem, after course, we used the Questionnaire for Assessment of Mental Health Literacy – QuALiSMental (Loureiro, 2015), adapted for three mental health problems (depression, schizophrenia and alcohol abuse).

Statistical analyses included descriptive statistics, Cochran Q test and Friedman test comparing data of the three mental health problems, but in some subject’s. For evaluate effect size, we used the ℜ measure.

**Results:**

In terms of results, it is observed, in the end of the intervention, high levels of MHL in all its components for depression, schizophrenia and alcohol abuse.

Comparing the three mental health problems, different results (p <0.05) were observed for each mental health problem presented, with high to moderate effect size measures.

The intention to seek help in mental health is comparatively higher in depression and alcohol abuse (p<0,05), as self-confidence for providing help (p<0,05). This pilot study demonstrated the potential for the MHFA program to improve mental health literacy associated to first aid.

## A21 Quantitative analysis of mammography units available in the Unified Health System in the states of Acre and Amapá

### Márcio T Oliveira^1^, Eduardo H Nakamura^1^, Murilo M Frigo^1^, Sofia U Frigo^1^, Samuel C Aragão^2^, Suellen M Oliveira^1^, Rogério A Antoniassi^1^, Douglas F Toledo^1^

#### ^1^Federal Institute of Education, Science and Technology of Mato Grosso do Sul (IFMS), Mato Grosso do Sul, Brazil; ^2^Federal Institute of Education, Science and Technology of Pará (IFPA), Pará, Brazil

##### **Correspondence:** Márcio T Oliveira (marciot2@gmail.com)

The breast cancer is a disease caused by the uncontrolled increase in breast cancer cells. In 2018, it was estimated that about 59.700 new cases of the disease appeared, with a significant pre-disposition in women (99%). The diagnosis of the disease can be made through medical examination or/and using the mammography. Currently, the mammography examination can be performed free of charge by the Unified Health System (SUS) of Brazil. However, many Brazilian cities do not have this equipment. The objective of this work is to perform a quantitative study of the equipment (mammography) available in the Unified Health System in the states ofAcre and Amapá. Data extraction was carried out using information available on the Brazilian government portal through the access to information program. The information was treated and analyzed using software for numerical processing. The state of Acre has twenty-two municipalities, of which only the cities of Cruzeiro do Sul and Rio Branco have available equipment, with two and ten apparatuses respectively. The state of Amapá has sixteen municipalities, of which Laranjal do Jari and Macapá have one and eleven mammography, respectively. From the analysis carried out, it is concluded that there is a considerable lack of mammography to assist in the early diagnosis of breast cancer in the states of Acre and Amapá. This research was supported by Federal Institute of Education, Science and Technology of Mato Grosso do Sul.

Keyword: Mammography, Breast Neoplasms, Demography Study

## A22 Social Network Analysis of patient movement for undergoing mammography examinations in the states of Acre and Amapá

### Márcio T Oliveira^1^, Douglas F Toledo^1^, Murilo M Frito^1^, Sofia U Frigo^1^, Eduardo H Nakamura^1^, Samuel C Aragão^2^, Rogério A Antoniassi^1^, Suellen M Oliveira^1^

#### ^1^Federal Institute of Education, Science and Technology of Mato Grosso do Sul (IFMS), Mato Grosso do Sul, Brazil; ^2^Federal Institute of Education, Science and Technology of Pará (IFPA), Pará, Brazil

##### **Correspondence:** Márcio T Oliveira (marciot2@gmail.com)

Currently in Brazil, mammography is considered the main imaging exam for the diagnosis of breast cancer. It was estimated that in 2018, about 59,700 new cases of the disease appeared, of which 99% were female. One of the major governmental challenges is the early diagnosis of breast cancer. Social Network Analysis is an important ally for network projection of graphs for visualization of information. The objective of this study is the construction of a network of graphs through the Social Network Analysis - SNA to present the population movement of patients for mammography in the states of Acre and Amapá. To extract the data, information was available through the government access portal through the information access program. The information was treated and analyzed using software for analysis of moving networks. Through the networks, patients were found to move to two cities: Cruzeiro do Sul and Rio Branco (the only ones with available mammography) in the state of Acre; and Laranjal do Jari and Macapá, both in the state of Amapá. It is concluded that the applied Social Network Analysis offers a broad visualization of the population displacement to perform the mammography exam, used for the diagnosis of breast cancer. This research was supported by Federal Institute of Education, Science and Technology of Mato Grosso do Sul.

**Keyword**: Mammography, Breast Neoplasms, Social Network Analyses

## A23 Palliative in intensive care in the perception of the nursing team

### Manuela G Borel, Thais N Faria, Fábio C Carbogim, Maira B Thofehrn, Jaqueline F Bittencourt, Thayenne B Monteiro, Ian C Toti, Danielle B Franck, Thais V Oliveira

#### Federal University Juiz de Fora, Juiz de Fora, Brazil

##### **Correspondence:** Manuela G Borel (mgcjfmg@yahoo.com.br)

**Introduction:** technological advances have enabled new care techniques to terminal patients die redirecting the process: before close to the family today in the health centers. This panorama motivates change care paradigm in terminally guided by the intervention to full shares. Although little known and played, palliative care is care alternatives to patients and families, terminally ill, through the prevention and relief of suffering. **Objective:** To know the perception and experience of the nursing team on palliative care for terminally ill patients. **Method:** qualitative study, conducted in hospital located in Brazil. Participants are 15 nurses and nursing technicians. Data were collected between July-August 2015 through open questionnaire, the interviews were recorded and transcribed. We used the operative proposal to Minayo, such as data analysis, in order to identify the senses cores to the analytical object (Minayo, 2014). **Results / Discussion:** the speeches emerged categories: (a) the perception and experience of the nursing team on palliative care; (B) as palliative care is applied; (C) working with families facing the terminal state. Below are the main stories in each category: (a) “understand palliative care as you keep watching, keep believing”; (B) “with the same care who have a chance of cure is with end-stage [...] shaven, leaving sanitized well”; (C) “a more serious patient I always ask for family if you have someone else who wants to see [...] the patient even being unaware that the family can talk to him [...] enhancing the contact. *”. Demonstrating that palliative care are to provide basic patient care, making use of technical and scientific skills combined with specific actions. **Conclusion:** to offer palliative care is to experience and share moments of faith, love and compassion, enabling die with dignity, accompanied by professional, family and spiritual support. However the study found that the nursing staff does not have a uniform knowledge, revealing weaknesses in the training process in palliative linking scientific knowledge to the relief of suffering.

**Keywords:** Palliative Care; Nursing; Hospice Care

**Reference**

1**.** Minayo, MCS. *O desafio do conhecimento: pesquisa qualitativa em saúde* (14a ed.). São Paulo: Hucitec, 2014, 1-406.

## A24 Nursing safe staff: Characteristics and impact on the quality and safety of care

### Maria João Freitas^1^, Pedro Parreira^2^

#### ^1^Nursing School of São Francisco das Misericórdias, Lisbon, Portugal; ^2^Health Sciences Research Unit: Nursing (UICISA:E) Nursing School of Coimbra, Coimbra, Portugal

##### **Correspondence:** Maria Freitas (mjbsfreitas@gmail.com)

**Background:** The Nursing Safe Staff (NSS) concept is complex, integrating characteristics that self-Influence. The literature recognizes the nurses number, with the appropriate competences as major characteristics, however, reveals inconsistency regarding its operationalization and influences at the quality and safety of nursing care provided.

**Objectives:** Identify the characteristics of the NSS and the results in the quality and safety of care.

**Methods:** Quantity, cross- sectional study with a sample of 628 nurses, 43 nurse chiefs, 1290 patients, of 43 units of internment, of 8 Portuguese hospitals. To collect information about “Availability of nurses in the appropriate amount”, “Availability of nurses with appropriate competencies” (Age, Total Professional Experience (TPE), Service Professional Experience (SPE), Initial /post graduate training (I/PGT) and “Risk and Occurrence Adverse Events in Patients” [3], two questionnaires were applied to nurses, and chief nurses. To know the patient satisfaction was used the scale “Patients Satisfaction with the Nursing Care”. For data analysis was used descriptive and inferential statistics with the Statistical *Software* SPSS®.

**Results:** Cluster analysis technique identified two clusters and variables that exerted [4] great influence on their constitution: “Age” (F=78,657); “TPE (F=74,626); “SPE” (F=49,520); “I/PGT” (F=66,157) and “Availability of nurses in the appropriate amount” (F=30,525). Two standards emerged: Standard A (55,8%) balanced teams, with amount nurses recommended by the Ministry of Health [5] or above, with greater maturity in terms of age, professional experience and more post graduate training; Standard B (44,2%) unbalanced teams, with nurses’ deficit’, and with less developed skills profile. The results of statistic test *t*-Student show averages of “Patients Satisfaction with the Nursing Care” significantly different in the two Standards (t (43) = 2,502, p=0,016), higher in services with dimensioning Standard A (AA =4,534). Also, patients cared for by teams of Standard B (AB =2,908) are more exposed at occurrence of adverse events associated with nursing practices (t (43) = - 2,771, p=0,008).

**Conclusions:** This study allowed to list the characteristics of the NSS important for its operationalization and the effect who exercise to the level of patient satisfaction and safety, assuming as a contribution to the informed decision of the nursing leaders.

**Keywords:** Health care allocation resources; Nursing human resources; Safe nursing supplies; Safe Staff Nursing; Patient satisfaction; Patient safety

## A25 Orthopedics and traumatology service: Adverse events

### Jessica C Santos^1^, Elenir P Paiva^1^, Fabiano B Loures^1^, Meire C Almeida^2^, Maria S Keulen^1^, Thainara L Silva^1^, Manuela G Borel^1^, Adriana E Oliveira^1^, Vanessa V Motta^1^, Danielle B Franck^1^

#### ^1^Federal University of Juiz de Fora, Minas Gerais, Brazil; ^2^Oswaldo Cruz Foundation, Rio de Janeiro, Brazil

##### **Correspondence:** Jessica C Santos (jessicacastroenf@gmail.com)

**Objective:** To identify the occurrence of adverse health events in an orthopedics and Traumatology ward.

**Method:** A retrospective review of medical Records was performed based on a simple random sample of 12 months, using the IHI Global Triggers tool to measure adverse events. Sample based on: 10% error, statistical significance of 95%, expected frequency of 10% and loss of 10%.

**Results:** A total of 322 records were evaluated during 1 year of study, 60 triggers were found and 19 adverse events were identified. There was a higher occurrence of adverse events per thousand patients per day in February (23.2%), March (20.0%) and may (16.1%), with an average of 1,000 hospitalizations per day of 13.9 and varying between 5.1 and 12.7%.. There were 4.15 adverse events for every hundred hospitalizations for one year.

**Discussion:** The indexes of occurrence of adverse events vary widely among different environments, although the same methodology is used for its detection. Most of the adverse events found are classified in category F, temporary damage, which required admission or prolongation of this category. Studies showed 96% and 53%. The Global Trigger Tool demonstrated great effectiveness in the search for adverse events during hospitalization of patients in the researched institution.

**Keywords:** Nurses; Environment; Environment and Public Health

## A26 Wellness and quality interpersonal relationships in the context of health care the nurse's perspective

### Sandra Queiroz^1^, Elsa Restier^1^, Luísa Ribeiro^2^, Odete Nunes^2^, Tito Laneiro^2^

#### ^1^Nursing School of S. Francisco das Misericórdias, Lisbon, Portugal; ^2^Autónoma University of Lisbon, Lisbon, Portugal

##### **Correspondence:** Sandra Queiroz (sandra.m.queiroz@gmail.com)

**Introduction:** The nursing skills plays a central role in the quality of care and it is essential to provide a quality service provided by dedicated and healthy nurses, which allows good performance.

**Objective:** To reflect, from the perspective of the nurse, on the conditions that affect the quality of interpersonal relationships and their consequences on the wellbeing and health of professionals.

**Method:** Qualitative study, exploratory and descriptive. A semi-structured interview with the feature analysis of content to a sample of 19 Nurses.

**Results:** Nurses (89% female; 11% male) with ages between 22 and 50 years; 5 of them with managerial functions and the remaining nurses play roles in internment services. They have between 1 and 23 years of professional activity, with experience between 6 months and 20 years in the hospital. Categories of analysis: empowerment; professional life; incivility, civility and engagement at work; burnout; health and wellbeing; affective organizational commitment; intention to quit. Nurses majority value healthy work environments which are promoters of personal development and professional effectiveness. The incivility is associated with the high workload, feelings of ineffectiveness and emotional exhaustion. The tiredness generates intention to change employment. They refer work engagement, with the detriment of health. Nurses recognize as being at the service of those who need to be cared, disparaging of their wellbeing and physical and mental health, working until exhaustion so they do not fail with their obligations. Emotional exhaustion is a reality expressed by these professionals, increasing with age and with the longest time of service.

**Conclusion:** There is incivility and conflicts in interpersonal relationships in services; the nurses who live in this reality suffer from emotional exhaustion and a perception of professional inefficacy, which are indicators of professional exhaustion. The quality of interpersonal relationships, the welfare and health of nurses is influenced by the high workload. In order to create appropriate interventions to promote good working conditions, health and wellbeing of nurses, it is essential to know the reality of each institution.

**Key Words:** Nurses; Health and Wellbeing; Burnout; Incivility; Workload.

## A27 Technology in health: The implementation of software for the management of the care of people with chronic diseases

### Karini Omena^1^, Wagner Marques^2^, Marcius Siqueira^3^, Marcos Santos^4^

#### ^1^State University of Health Sciences of Alagoas, Maceió, Brazil; ^2^PGS Medical, Penedo, Brazil; ^3^City Hall of Penedo, AL, Penedo, Brazil; ^4^Science and Technology’s Institute of Penedo AL, Penedo, Brazil

##### **Correspondence:** Karini Omena (karinimenezes@yahoo.com.br)

**Background**

The Unified Health System (SUS), which is based on Primary Health Care (PHC), make a close relationship between improving the quality of care, efficiency and lower costs.

In this context there is the configuration of the Health Care Network of People with Chronic Diseases, as it is a serious public health problem and needs specific care.

Chronic diseases have strong impacts on individuals as they have adverse effects on quality of life, cause premature deaths, and generate negative economic implications for families, communities and countries (MENDES, 2011).

**Goal**

This work’s purpose is to show a technological increment used in the management of health care of patients with chronic diseases in the scope of Primary Care.

**Methods**

In 2018, Penedo/AL city implemented a software developed by the companies PGS Medical and NAGIS Health using artificial intelligence algorithms for the development of health care protocols called Highly Resolutive Center - HRC.

The program is developed with software that has diagnostic and therapeutic protocols, which manage the care from the orientation of the professional's work schedule, to the indicative of changes in therapeutic project goals, according to the degree of complexity, allowing the visualization of the trajectory and the new flows to be traced in the care line. Individual monitoring and evaluation of the monitored patients are also analyzed through the software, measuring results in terms of quality of life, as well as the economic result for the city.

The pacient also has at his disposal a central telephone answering service, where doctors, with access to the software, can guide the patient about palliative care, medication, referrals and other care.

**Result**

The experience in the city has been successful, with results of 90% reduction in the number of entries of the accompanied patients with the use of the software in the Pronto Atendimento service and a 56% reduction in hospitalizations in hospital beds.

**Conclusion**

Providing tools and technological resources for the AB teams’ work constitutes an important strategy for the qualification and effectiveness of health care, avoiding high costs and little resolution to their health problems and consequently low quality of life.

**Keywords**: Health Technology, Chronic Disease, Quality of Life

**Reference**

Mendes, EV. As redes de atenção à saúde. Brasília: Organização Pan-Americana da Saúde. Brasília, 2011, 1-554.

## A28 Nurse’s skills as facilitator on the family conferences in palliative care

### Ivo Paiva, Ana Rocha

#### Portuguese Oncology Institute of Coimbra, Coimbra, Portugal

##### **Correspondence:** Ivo Paiva (ivocsoarespaiva@gmail.com)

**Background**: In Palliative Care (PC), the patient/family should be integrated in the discussion and execution of the advanced care plan1. In this context, the Familiar Conference (FC) appears as a structured meeting, that promotes the communication between the healthcare team and the patient/family. The FC has guidelines of good practice. Reviewing the literature, one can verify that the healthcare teams don't follow a structured model and the nurse have skills to facilitate their execution.

Objectives: Characterize the structures of the FC in PC; identify the skills of the nurse in the FC.

**Methods**: An exploratory study, quantitative, observational, analytic and transversal, conducted from February 6th to April 6th of 2017 in four units of PC, in an accidental non-probabilistic sample. Application of the form of “Characterization of FC”, created and submitted to facial and content validation, by FC and PC experts. The study was approved by the ethic committees of the referred institutions.

**Results**: 94 FC were analyzed. 90,4% of the FC took between 18 and 54’, with a median of 3 professionals and 2 family members present. The patient was present in 36,2% of the FC. In the FC characterization, one can identify that: 87,2% were planned and 62,8% took place in a reserved room/cabinet. In average, 3 objectives were addressed and in 84% there was consensual decision-making. The nurse was the professional more present, being in 98,2% of the FC; managing 60% of the cases, increasing the presence of members of the multidisciplinary team. As well, the nurse potentiated the holistic approach of the patient/family in the physical, psychosocial, spiritual and economic dimension, which is less observed in the remaining managers (doctor, psychologist and social assistant).

**Conclusions**: The FC is a method that enhances the relation between the healthcare professional and the patient/family, which may contribute in PC to optimize the quality of the care provided, hence we recommend its integration in the caring process. By being the professional more present and the responsible for the FC, the nurses have communication, relational and leadership skills that legitimize them to be the facilitator of the FC.

## A29 A study of pleasure and suffering in the work of nurses employed in primary health care in a municipality in the hinterland of Minas Gerais state, Brazil

### Bruno A Ribeiro^1^, António F Teixeira^2^, Silvia S Silva^3^

#### ^1^University Center of Formiga (UNIFOR-MG), Minas Gerais, Brazil; ^2^University Center Senac, São Paulo, Brazil; ^3^Ribeirão Preto University (UNAERP) Campus Ribeirão Preto e Guarujá, Ribeirão Preto, Brazil

##### **Correspondence:** Bruno A Ribeiro (br.alvarenga@yahoo.com.br)

**Introduction:** There is rich literature on pleasure and suffering in the work of nurses. Suffering is usually linked to strenuous daily hours and work overload, inadequate remuneration and sources of tension in health care jobs. Pleasure is associated with the professional recognition that originates in manifestations of gratitude for the rendered services in addition to caring itself, which brings the feeling of duties being accomplished. This study took as a relevant object of study the situations that can cause pleasure and pain in the work of nurses who are employed in primary health care in a municipality of the state of Minas Gerais, Brazil.

**Objective:** Explain how situations leading to pleasure and pain at work relate to how nurses employed in primary health care in the municipality of Formiga/MG, Brazil, express their subjectivities in the socio-occupational space in which they are inserted.

**Methods:** This is a qualitative, exploratory-descriptive study carried out in 2018, following approval by the Research Ethics Committee, with 17 nurses with managerial positions in Family Strategy Units in the municipality under study. Interviews have been conducted to collect data which were then subjected to content analysis.

**Results and Conclusions.** Poor working conditions appear as one of the main causes of suffering at work, which is linked to the decaying standards of health care in Brazil. Nurses deal with suffering in different ways, especially with the strengthening of interpersonal relations with their peers and the setting up of a group that seeks to foster continuing education, thus representing a political and psychological instrument. The most common sources of pleasure are the recognition from health care users, their recovery, and the possibility of helping them and establishing closer contact with them. It can be concluded that the strategies used to deal with suffering and to offset them with experiences that generate pleasure represent special means for the expression of subjectivity of nurses in dealing with their work. Further studies are recommended, including other professional groups, in the same municipality or other locations, in primary health care, with a view to generating a more profound understanding of the theme.

**Keywords:** Nurses. Primary health care. Work conditions.

## A30 Market implementation and end users’ adoption of Ambient Assisted Living solutions: A literature review

### Soraia Teles^1^, Ana M Ferreira^2^, Diotima Bertel^3^, Rita T Sousa^4^

#### ^1^Institute of Biomedical Sciences Abel Salazar, Department of Behavioral Sciences, University of Porto & Center for Health Technology and Services Research (CINTESIS); ^2^Faculty of Medicine University of Porto & Center for Health Technology and Services Research, Porto, Portugal; ^3^SYNYO GmbH, Vienna, Austria; ^4^Department of Behavioral Sciences, University of Porto & Center for Health Technology and Services, Porto, Portugal

##### **Correspondence:** Soraia Teles (teles.s.soraia@gmail.com)

**Background**: The Ambient Assisted Living (AAL) concept corresponds to a new paradigm, building on the potential of ubiquitous ICT devices and new forms of interaction to improve older adults’ health, autonomy, social integration and quality of life. In spite of the growing importance of AAL solutions for independent ageing, a gap between technology development and its adoption by end users has been reported in the literature. This study aims to provide a comprehensive overview on factors i. supporting or hindering the adoption of AAL solutions by primary and secondary end users, i.e. older adults and informal/professional caregivers; and those ii. supporting or hindering the implementation of AAL solutions in the market by their providers or enablers.

**Methods**: A narrative literature review on research findings published between 2007 and 2017 was carried out. It was organized in three operational stages: i. definition of search objectives, keywords, databases and inclusion/exclusion criteria; ii. papers gathering and multistage filtering; and iii. content analysis and interpretative qualitative synthesis of the search results. After the removal of duplications and application of inclusion/exclusion criteria, seventy-nine papers were included in the analysis. Resourcing to a qualitative method of thematic coding, factors identified in the papers as supporting or hindering the adoption of AAL solutions by primary and secondary end users, and the implementation of AAL solutions in the market by their providers or enablers, were extracted and categorized.

**Results**: Perceived usefulness, self-efficacy, and perceived impact of AAL technologies on older adults’ social interaction and self-image emerged as the most reported attitudinal factors influencing AAL solutions’ adoption by primary and secondary end users. Most identified technical factors were i. system’s usability; ii. security, confidentiality and privacy; iii. reliability, accuracy; and iv. customization features. For providers and enablers, a successful AAL implementation seems to be determined by factors related to the existence/inexistence of collaborative businesses ecosystems, prevailing funding and reimbursement mechanisms as well as market regulation.

**Conclusions**: The uptake of AAL solutions by end users depends on the interaction between technical and attitudinal factors. The effective implementation of AAL solutions in the market relies heavily on successfully establishing stakeholder collaboration.

**Key Words:** Ambient Assisted Living (AAL); Healthy Ageing; Ageing in Place; Technology Adoption; Multi-stakeholder Perspective.

## A31 Short-term face-to-face and online psychodynamic psychotherapy: The perception of patients

### Fernando L Macedo^1^, Edilson C Carita^2^, Silvia S Silva^2^, Renata P Clemente^1^, Loiane L Santos^1^, Randolfo S Junior^3^

#### ^1^Municipal Institute of Higher Education, Ribeirão Preto, Brazil; ^2^University of Ribeirão Preto, Ribeirão Preto, Brazil; ^3^Faculty of Medicine of São José de Rio Preto, Brazil

##### **Correspondence:** Fernando L Macedo (fernando.planetasurf@gmail.com)

**Introduction:** In the last decades multiple activities via the internet have been growing exponentially, and online psychotherapy is following this trend. It is thus important to recognize this form of work facilitation for people in today’s world. In Brazil, psycotherapy over the internet is permitted by the Federal Psychology Council only on an experimental basis, for instance research. In this study, Therapeutic Alliance has been considered as an instrument to construct the patient-therapist bond.

**Objective:** Compare the perception of patients who have undergone short-term face-to-face and online psychodynamic psychotherapy, with a single therapist.

**Methods:** This is a qualitative, exploratory-comparative study that deployed a clinical methodology. The treatment was systematic and respective, comprising six daily face-to-face sessions in consecutive days, except Sundays; and six online sessions using the same data collection procedures, each session lasting for between 40 and 50 minutes. For the collection of data on the perception of patients about the treatment – face-to-face versus online short-term psychodynamic psychotherapy – the Working Alliance Inventory was used with questions concerning the therapeutic relation after the 6 online sessions, and the Session Evaluation Questionnaire immediately after the end of each psychotherapeutic session, both face-to-face and online*.*

**Results and Conclusion:** The findings show face-to-face and online treatments to be equivalent as regards the therapeutic relation established at both moments. It has also been concluded that the setting created by online treatment, no longer face to face in the traditional setting, has not been perceived as something different by patients; and, on some occasions, the online treatment has been preferred especially due to convenience. However, in this study, one reason for the satisfactory results favoring online psychotherapy may have been that face-to-face treatment was provided firstly, which may have produced a more favorable therapeutic alliance through positive transfer, later favoring an online treatment that produced stronger bonds, without the occurrence of therapeutic impasses and resistance.

**Keywords:** Short-term psychodynamic psychotherapy. Therapeutic Alliance. *Internet.Online.*

## A33 Challenges for and contributions of psychologists regarding bereavement within hospitals

### Fernando L Macedo^1^, Walison H Tozatto^1^, Therezinha A Bochio^1^, Loiane L Santos^1^, Mariana A Porto^1^, Edilson C Caritá^2^, Silvia S Silva^2^

#### ^1^Municipal Institute of Higher Education IMES Catanduva, SãoPaulo, Brazil; ^2^Ribeirão Preto University, Ribeirão Preto, Brazil

##### **Correspondence:** Fernando L Macedo (fernando.planetasurf@gmail.com)

**Introduction:** From ancient times the relation of humans with death has been permeated by a mixture of fascination and anguish. With the advances in medicine and technologies in the health sciences, finitude has become part of hospital complexes, thus causing bereavement to become institutionalized. Psychologists who routinely follow patients in serious conditions and their families need qualification, technical training and emotional competencies.

**Objectives:** Understand the role of psychologists in caring for patients experiencing bereavement and describe the sociodemographic profile of such professionals.

**Methods:** This is a quali-quantitative, descriptive study, in which psychologists working in hospitals were invited to participate. The data were collected by means of interviews and a sociodemographic questionnaire. Descriptive statistics was used to analyze quantitative data, and phenomenological analysis to deal with qualitative data following Amatuzzi’s approach (2009).

**Results and Conclusion:** The sample was comprised of 6 psychologists, at an average age of 32.6 years; most of them spiritualist (50%) and single (83.3%); they indicated they were happy in their jobs; however, 66.7% were not undergoing psychological monitoring at the time. Among meaning categories, the following have stood out: cultural aspects related to death and dying, the work with families, the role and the emotional issues of professionals, and unacknowledged grief. The participants pointed out the following contributions of psychologists in hospital routines: interventions to relieve the pain and suffering of patients, families and teams, seeking alternative treatments for individuals or groups. They also underscored that challenges for their professional work involve recognition of their professional roles by teams in addition to gaps in their education/training, considering that curricular components do not cover specificities for dealing with death. In this regard, this study is expected to point at the relevance of professional self-care, through psychotherapies, supervision and group support to seek alternatives that may enable them to deal with the emotional stress of the job.

**Keywords:** Bereavement; Psychology; Hospital

## A34 The perception of health-care professionals regarding the birth plan's use

### Dolores Sardo^1^, Arminda Pinheiro^2^

#### ^1^Nursing School of Porto, Porto, Portugal; ^2^Nursing School of Minho University, Braga, Portugal

##### **Correspondence:** Dolores Sardo (dolores.sardo@gmail.com)

**Introduction:** The Birth Plan (BP) is a written document prepared by the couple to express their wishes regarding childbirth1,2,3 Although there is a shortage of research in Portugal4, it maintains a focus in healthcare.

**Objective:** To identify the perception of health-care professionals regarding the birth plan in Portugal.

**Method:** Qualitative, exploratory, descriptive study. Using self-filling questionnaire online, between April to May 2018. Anonymity and confidentiality was assured. In the statistical treatment we used the content analysis according to Bardin5 and the tool INVivo/12. A no probabilistic, intentional sample, n = 188, 93.1% female, x = 44.2 years, 71.0% nurses midwives that work in delivery rooms (54.8%).

**Results:** The health-care professionals associated BP following descriptors: empowerment and decision, humanization, choice and respect. 67.0% informed the woman/couple about BP in prenatal appointments and in the childbirth education class. 44.1% assisted in their construction in prenatal appointments and in the childbirth education class; 44.6% reported difficulties due to lack of knowledge/information of women/couples, lack of adequacy between expectations/choices of women and the availability of resources focusing the choices made by the couple, lack of empowerment of women/couples, fear the professionals reactions. 93.1% considered important to introduce BP in the health-care team before delivery to attending the possibility of the couple’s choice. 65.4% felt that the BP was not respected in the maternities due to institutional and professional reasons. 89.9% thought that the health-care professionals had differing reactions to the BP, ranging from devaluation / disrespect to attitudes of acceptance and openness. The majority mentioned advantages in the utilization of the BP for both, the healthcare team and the woman/couple, suggesting that it should be a single and mandatory document because it focuses care on couples, increases the empowerment and satisfaction of the woman/couple, obstetric violence and overuse of medicalization.

**Conclusions:** The results show that the health-care professionals recognize the advantages of the BP as a facilitating and reorienting tool of the woman/couple at birth, which promotes a respectful and client-centered care. The BP continues to be incipient practice in Portugal. The health-care professional suggests measures and policies to promote BP implementation.

**Keywords:** Birth plan; health-care professionals; perception

## A35 An outline of service and embracement in spontaneous demands using the classification of alert signs in a Basic Health Care Unit of Ribeirão Preto, Brazil

### Silvia S Silva, Gabriela B Pegoraro, Rosemary A Daniel, Edilson C Caritá

#### Ribeirão Preto University, São Paulo, Brazil

##### **Correspondence:** Silvia S Silva (sssilva@unaerp.br)

**Introduction:** Primary Care is regarded as the “main entrance” to health care, from which point other relations are established with intermediate and high complexity health care. Thus, Basic Health Care Units (UBS) have to ensure access and embracement to citizens within a rationale of organization and effectiveness of the work. In order to organize the forms of admission for users to the municipal network of Primary Care in a municipality in the hinterland of São Paulo state users can, according to the classification of alert signs (using red, yellow and green color tags), receive immediate, priority or on-the-day care, scheduled care between 1 and 7 days, or routine scheduled care – respectively; their symptoms being relieved, in addition to guaranteed continuity of ambulatory treatment.

**Objective:** Describe the characteristics of service and embracement of spontaneous demands in a UBS of São Paulo hinterland, Brazil.

**Methods:** This is a descriptive, cross section, quantitative and retrospective study using documental research. 1,416 records of health care service were analyzed that had been recorded in the Embracement Form for Spontaneous Demands, 409 of which involved pediatrics, and 905 internal medicine, over a period of eight months in 2016.

**Results and Conclusion:** In the UBS catchment area, the most prevailing classification was green, with 49.58% scheduled treatments for 1 to 7 days; most demands related to women above 20 years of age (44.5%); in the morning (57.89%); predominantly on Mondays (23.73%), as happens in the Emergency Health Care units in the municipality. We conclude that, in the studied UBS, the spontaneous demand does not show critical clinical conditions during embracement and the classification of alert signs, thus it requires guidelines to deal with the flow and norms for this level of health care service; the demand by women confirms their propensity to seek health care spontaneously, as well as a different perception of their health-illness processes. The study was presented to the UBS health care team and local managers in the municipality to increase sensitivity to the use of this kind of embracement with the population, in a more assertive and safe manner.

**Keywords:** Primary Health Care. Embracement of spontaneous demands. Vulnerability.

## A38 Using face recognition for emergency sharing of e-health records

### Jose García-Alonso^1^, Javier Berrocal^1^, Jaime Galán-Jiménez^1^, Juan M. Murillo^1^, David Mendes^2^, César Fonseca^2^, Manuel Lopes^2^

#### ^1^University of Extremadura, Extremadura, Spain; ^2^Nursing School of Évora, University of Évora, Évora, Portugal

##### **Correspondence:** Jose García-Alonso (jgaralo@unex.es)

**Introduction:** As technology advance, more and more health information is available through electronic means. Between the many uses of this information, emergencies are a great example in which the pervasive presence of e-health records can have a significant impact. However, the personal nature of this records and the privacy concerns they raise can complicate its use in emergency situations.

**Goals:** In this work we propose a mobile application based on face recognition to share health information. The proposed solution is designed to work by proximity, so privacy is reinforced while making the needed information available to nearby professionals.

**Method:** We have developed a mobile application that stores the relevant health information on the patient smartphone. The user can control the availability of this information by only making it accessible to other devices after they complete a positive face recognition. Face recognition is performed using image recognition techniques on the smartphone of the user asking for health information. If the result is positive, the health information is transmitted directly between the devices, without being uploaded to the internet.

**Results:** The presented application is based on the use of state-of-the-art face recognition algorithms, and therefore, perform this task with high precision. Using this kind of authentication, and using proximity based communications technologies like Bluetooth or Wifi, a privacy aware sharing of e-health records is performed. This solution is designed to be used in emergency situations where the patient cannot personally grant access to his/her information.

**Conclusions:** The mobile application presented in this work, allow patients to share their medical information in case of an emergency. There is no need for the user to directly grant access to this information, so it can be used during emergencies. However, only nearby devices who performed a positive facial recognition could access the information which provides an improved privacy and security control over the health information.

**Keywords:** Face recognition; eHealth records; Mobile computing

## A40 Home support in older adults in the community

### Maria Cristina Faria, Xavier Balancho

#### Polytechnic Institute of Beja

##### **Correspondence:** Maria Cristina Faria (mcfaria@ipbeja.pt)

**Background:** Evidence has projected that the proportion of people aged 65 and older is forecast to almost double between 2010 and 2050 and the number of people aged 85 years and older is projected to rise from 14 million to 19 million by 2020 and to 40 million by 2050. In order to meet the needs of this population, diversified services arise to protect their health and well-being in place. Home support is one of the structures to support ageing place in the community. The aim of this study is to analyze mental health, well-being and life satisfaction in old adults that resort to the service of home support.

**Methods:** Qualitative and quantitative methods were used involving interviews and instruments of psychological health assessment with older adults (n = 10). Five are clients of a private company and five of a private social solidarity institution. Data were analyzed thematically and with descriptive statistics.

**Results:** Findings show that the participants are satisfied with the home support. The users of the institution nevertheless claim that this service should be more extensive. The company's customers claim that the service is expensive. When applying the instruments of mental health and the quality of life assessment it was identified a better mental health and life satisfaction in the company's clients.

**Conclusions:** According to the results, intervention strategies were designed in order to streamline the social response of home support. It is important to develop participatory approaches to engage the older adults in healthcare at home support, in order to ensure that services are relevant to these groups. The research made possible a better understanding of the specificity of home support and helps to reflect on intervention strategies in this area. From this information, a proposal for an intervention project called “At home is possible!” was drawn up. The project of intervention also aimed at a perspective of joint intervention, considering three aspects: older adults, family and community.

**Keywords:** Older adults, Home support, Ageing in place, Mental health, Well-being, Life satisfaction, Interventions.

## A43 Innovative devices for physical rehabilitation in bedridden elderly patients: A scoping review on key characteristics

### Rafael Bernardes, Arménio Cruz, Anabela Salgueiro-Oliveira, Paulo Costa, João Graveto, Liliana B. Sousa, Pedro Parreira

#### Health Sciences Research Unit: Nursing (UICISA:E) Nursing School of Coimbra, Coimbra, Portugal

##### **Correspondence:** Rafael Bernardes (rafael.alvesbernardes@gmail.com)

**Introduction:** The loss of muscle mass is a normal physiological alteration that results from the aging process. During acute conditions, where institutionalization is necessary, or due to specific chronic diseases, bed rest can be advised as a therapeutic measure. However, extended periods (>10 days) of bed rest lead to significant changes in the body composition, resulting in metabolic/functional decline. In some settings, due to the lack of clinical staff, the complexity of care and inherent workload or due to the lack of motivation of patients/families, physical exercise is difficult to perform while bedridden. These realities can lead to negative health and well-being-related outcomes for the aged, such as sarcopenia and frailty. Therefore, innovative devices that aid bedridden patients to perform physical activities independently, following a plan previously delineated by health professionals, are an important way to prevent complications related to bed rest.

**Objectives:** To map all the patented European physical rehabilitation devices for bedridden patients and highlight their key characteristics.

**Method:** A scoping review was conducted. The search strategy included patents and prototypes registered in the European Patent Office between 2014 and 2018. Two independent reviewers analyzed the relevance of the findings and extracted and synthesized data.

**Results:** Sixteen rehabilitation devices were included, five being only indicated for lower limb rehabilitation. Regarding their structure and assemble: 6 are modular and require previous assemble; 2 devices possess an air pump; 6 display a sliding feature allowing a wider range of motion; 3 possess a mechanism that allows them to exercise in vertical motion; 2 can mobilize the patients limbs through a powered system (motor); and 1 device has a video camera that allows to record/monitor the patient’s progress. The devices present variable height and weight, mainly adjusted to a standard hospital bed dimensions.

**Conclusions:** A significant number of patented devices for the physical rehabilitation of elderly bedridden patients were found. The majority of these devices work upper and lower limbs through a rigid structure. Nonetheless, the use of technological advances (air pumps, sliders, motors, cameras) potentiates the development of innovative devices that foster a safer and more efficient rehabilitation intervention.

**Keywords:** self-help devices; exercise; bed rest; rehabilitation; aged; aged, 80 and over

## A47 Technology in the practice of nursing: Search for evidence

### Cláudia M Messias^1^, Elaine C Baptista^2^, Karen C Cítera^2^, Cláudia S Medeiros^2^, Ana K Brum^1^, Daniele M Silva^1^

#### ^1^Federal Fluminense University; ^2^Castelo Branco University

##### **Correspondence:** Cláudia M Messias (marimessi1512@gmail.com)

**Introduction**: Technologies assist in the attention of population health care. With the apparition of the technologies and their increasing insertion in the work process, requiring of the health professionals in particular the nursing team the knowledge and exercise of their practice. It is recommended a broad observation, taking into account the scientific advance, adding the conception of new knowledge, that allow transformations and quality in the work process. The nursing professional has been provided with foundations on this subject, in order to obtain competence to unfold modes of utility of health technologies. The nursing team in providing health care has three types of technologies: hard (equipment and machines); light (communication); hard-light (structured scientific knowledge). As a research question: What is the scientific production about the use of technologies in nursing work? With the objective of exploring the scientific productions that approach the use of technology in nursing work.

**Methodology**: Integrative review of the literature based on Health Science Descriptors (DecS): “Nursing”, using as search strategy the Boolean operators “and” for “technology” and “work”. The databases selected for research (BDENF) and Latin American and Caribbean Literature in Health Sciences (LILACS).

**Results**: The sample emphasizing technologies in nursing work with temporal cut of 05 years (2013-2018), were found in the following LILACS libraries: 688 studies, BDENF: 112 studies being 787 excluded because they did not meet the eligibility criteria, totaling the final sample of 13 articles. From the detailed reading of the evidence, two categories emerged: technology in the work process of the nursing team and technology in knowing and doing nursing. Nursing professionals, who have in their reality, the technological evolution, tend to be scientifically prepared, enabling the development of skill, competence to proceed under the humanized and interdisciplinary paradigm.

**Conclusion:** It was concluded that the understanding of the available technological tools and their management, it is indispensable for it to result in a care of excellence. The nursing professional who has his domain, becomes the protagonist of responsible and safe care.

## A56 Effectiveness of long-term health care in home context versus institutional care for the elderly dependent

### Susana Fonseca-Teixeira^1^*, Pedro Parreira^2^, Maria João Freitas^3^, Lisete Mónico^4^, Lorenzo Mariano^5^, Jose García-ALonso^5^, João Amado^1^

#### ^1^Health Sciences Institute, Catholic University of Portugal, Porto, Portugal; ^2^Health Sciences Research Unit: Nursing (UICISA:E) Nursing School of Coimbra, Coimbra, Portugal; ^3^Nursing School of São Francisco das Misericórdias, Lisbon, Portugal; ^4^University of Coimbra, Coimbra, Portugal; ^5^University of Extremadura, Extremadura, Spain

##### **Correspondence:** Susana Fonseca-Teixeira (susanaalexandra.t@gmail.com)

**Introduction:** Demographic aging and changes in the epidemiological pattern have led to an increase in health expenditures, particularly long-term care. In this way, home support is pointed out as an important strategy with added benefits.

**Objective:** This review aims to identify and synthesize scientific evidence on the health gains of long-term care in a home context versus institutional care for dependent elderly people.

**Method:** A review of the literature published in the last 10 years in the EBSCOhost database was carried out in three languages (Portuguese, English and Spanish). The descriptors of Medical Subject Headings used were: “Nursing care”, “Health Services Accessibility”, “Long-term care”, “Home care services”, “At-home”, “Nursing home”, “Cost benefit analysis” and “Efficiency”.

**Results:** The results indicate that home-based care may be a lower cost alternative than institutional long-term care. The focus on home-based care is also linked to improved quality of care, lower access inequalities, more equitable and accessible care, reflecting not only the health outcomes of the population but also a reduction in the total cost of health care, resulting in affordable and sustainable health outcomes. Evidence has shown that interventions by multidisciplinary teams are more efficient and home-based care users have improved health status, lower admission rates to the emergency department, faster recovery and less likely to be hospitalized for adverse events, cutaneous infections and pressure ulcers. The empowerment of patients and caregivers in the care transition has also been shown to be an effective response in reducing re-hospitalization rates, reflecting lower hospital costs. On the other hand, studies indicate that the cost-effectiveness of home-based care is more effective only for less dependent elderly people and that they have access to informal caregivers.

**Conclusion:** Cost containment of institutional care and investment in home-based care are major challenges in increasing the efficiency of long-term and continuing care expenditures as well as greater partnership with social services, allow citizens to access the care they need more quickly.

**Keywords:** Nursing care, Long-term care, Cost benefit analysis, Efficiency.

## A57 Emotional intelligence, psychological capital, and organizational spirituality and in health organizations

### Lisete Mónico^1^, Cristina Falcão^2^, César Fonseca^3^, Manuel Lopes^3^, Lorenzo Mariano^4^, Jose García-Alonso^4^, Pedro Parreira^2^

#### ^1^University of Coimbra, Coimbra, Portugal; ^2^Health Sciences Research Unit: Nursing (UICISA:E) Nursing School of Coimbra, Coimbra, Portugal; ^3^University of Évora – Nursing School of Évora, Évora, Portugal; ^4^University of Extremadura, Extremadura, Spain

##### **Correspondence:** Lisete Mónico (lisete.monico@fpce.uc.pt)

**Introduction:** In the current economic environment, with regard to the human resources dimension, several studies show that a positive psychological capital assumes a preponderant role for the organizational practice, contributing to an increase of the productivity of the organization, as well as to a greater satisfaction of its collaborators.

Several studies have also shown that the spiritual characteristics of the organizations (elevation of organizational values) and the emotional intelligence of the employees positively impact on the psychological capital of the employees, through various indicators such as productivity or satisfaction and involvement/commitment with work.

**Aim:** We intend to evaluate the relationship and contribution of organizational spirituality and emotional intelligence in the positive psychological capital of organizations, starting from the research question: “What is the impact of emotional intelligence and organizational spirituality on the development of the psychological capital of employees in different organizations?”

**Method:** A cross-sectional study was used, comprising a sample of 874 workers from different organizations (154 from health). A questionnaire was applied, including three scales: Emotional Intelligence Scale (Rego, Sousa, Cunha, Correia, & Saur-Amaral), Psychological Capital (Luthans, Youssef, & Avolio), and Organizational Spirituality scale (Rego, Souto, & Cunha).

**Results:** The results suggest the existence of a high positive relationship between emotional intelligence, organizational spirituality, and psychological capital. Hierarchical multiple regression analysis allowed us to identify that psychological capital is predicted by Emotional Intelligence in 35%, specifically due to the factors “Understanding of own emotions”, “Self-control in the face of criticism”, “Self-encouragement”, and “Emotional Self-control, after controlling for sociodemographic variables (4% of explained variance, mainly due to the dummy variable performance of management functions).Organizational Spirituality was also a predictor of workers’ psychological capital, adding 12% of the explained variance to the previous regression model, due to the factors “Joy at work” and “Opportunities for the inner life”.

**Conclusion:** Organizational spirituality and emotional intelligence explained 51% of workers’ psychological capital, after controlling for performance of management function. The implications of the results obtained in this research are important in the selection, recruitment and training of care professionals in health organizations.

**Keywords:** Psychological capital, Emotional intelligence, Organizational spirituality, Health organizations.

## A58 Innovative devices for physical rehabilitation in bedridden patients: Current functional utility models’ characteristics and features

### Rafael Bernardes^1^, Arménio Cruz^1^, Anabela Salgueiro-Oliveira^1^, Paulo Costa^1^, João Graveto^1^, Liliana Baptista^1^, José García-Alonso^2^, Pedro Parreira^1^

#### ^1^Health Sciences Research Unit: Nursing (UICISA:E) Nursing School of Coimbra, Coimbra, Portugal; ^2^University of Extremadura, Extremadura, Spain

##### **Correspondence:** Rafael Bernardes (rafaelalvesbernardes@esenfc.pt)

**Introduction**: The loss of muscle mass is a normal physiological alteration that results from the aging process. During acute conditions, where institutionalization is necessary, or due to specific chronic diseases, bed rest can be advised as a therapeutic measure. However, extended periods (>10 days) of bed rest lead to significant changes in the body composition, resulting in metabolic/functional decline. In some settings, due to the lack of clinical staff, the complexity of care and inherent workload or due to the lack of motivation of patients/families, physical exercise is difficult to perform while bedridden.These realities can lead to negative health and well-being-related outcomes for the aged, such as sarcopenia and frailty. Therefore, innovative devices that aid bedridden patients to perform physical activities independently, following a plan previously delineated by health professionals, are an important way to prevent complications related to bed rest.

Objective(s): To map all the patented European physical rehabilitation devices for bedridden patients and highlight their key characteristics.

**Method**: A scoping review was conducted. The search strategy included patents and prototypes registered in the European Patent Office between 2014 and 2018. Two independent reviewers analyzed the relevance of the findings and extracted and synthesized data.**Results**: Sixteen rehabilitation devices were included, five being only indicated for lower limb rehabilitation. Regarding their structure and assemble: 6 are modular and require previous assemble; 2 devices possess an air pump; 6 display a sliding feature allowing a wider range of motion; 3 possess a mechanism that allows them to exercise in vertical motion; 2 can mobilize the patients limbs through a powered system (motor); and 1 device has a video camera that allows to record/monitor the patient’s progress. The devices present variable height and weight, mainly adjusted to a standard hospital bed dimensions.

**Conclusions**: A significant number of patented devices for the physical rehabilitation of elderly bedridden patients were found. The majority of these devices work upper and lower limbs through a rigid structure. Nonetheless, the use of technological advances (air pumps, sliders, motors, cameras) potentiates the development of innovative devices that foster a safer and more efficient rehabilitation intervention.

**Keywords:** self-help devices; exercise; bed rest

## A59 The impact of organizational practices and psychological empowerment on the behaviors of mobilization, job satisfaction and nurses’ turnover: An empirical study carried out in hospital

### João Moreira^1^, Pedro Parreira^2^, Lisete Mónico^3^, Rogério Ferrinho^4^, César Fonseca^5^, José García-Alonso^6^, Henrique Oliveira^4^, João Graveto^1^

#### ^1^Portuguese Institute of Oncology Coimbra, Portugal; ^2^Health Sciences Research Unit: Nursing (UICISA:E) Nursing School of Coimbra, Coimbra, Portugal; ^3^University of Coimbra, Coimbra, Portugal; ^4^Polytechnic Institute of Beja, Beja, Portugal; ^5^University of Évora – Nursing School of Évora, Évora, Portugal; ^6^University of Extremadura, Extremadura, Spain

##### **Correspondence:** João Moreira (ajoaomm@gmail.com)

**Aims:** The present study was designed to analyse the relationship between mobilization behaviours, psychological empowerment, practices of work organization, work satisfaction and turnover. The main psychometric properties of the self-reports instruments were also analyzed.

**Methods:** The sample consist of 338 nurses from Hospital and University Center of Coimbra. Regarding the evaluation instruments, a self-report questionnaire was used for the characterization of the sample, as well as four sections of the Nursing Questionnaire (Psychological Empowerment, General Job Satisfaction, Turnover and Mobilization Behaviours).

**Results:** We found an internal consistency higher than .60 for the four sections of the Nursing Questionnaire. The same trend is observed in the factorial analysis, which presents, in the same sections, good values for Kaiser-Meyer-Olkin measure and in the Bartlett’s test of sphericity. In the study of correlations, we observed that these are statistically significant on the variables between mobilization behaviours, psychological empowerment, practices of work organization and general work satisfaction. These results are consistent thourgh the analysis of multiple linear regression, where it is verified that, with the exception for turnover, all variable, individually, have a predictive value for mobilization behaviours.

**Conclusions:** It was verified that, with the exception of the turnover variable, the variables psychological empowerment, practices of work organization and general work satisfaction are related and have a predictive vale for mobilization behaviours.

**Keywords:** Nursing, mobilization behaviours, psychological empowerment, job satisfaction, practices of work organization, turnover.

## A60 Socio-demographic profile of people aged 65 and over in Portugal long-term care

### Ana Ramos^1^, Adriana Henriques^2^, Manuel Lopes^3^, Lisete Mónico^4^, Anabela Salgueiro-Oliveira^5^, Pedro Parreira^5^, César Fonseca^3^

#### ^1^Nursing School of Lisbon, University of Lisbon, Lisbon, Portugal; ^2^Nursing School of Lisbon, Lisbon, Portugal; ^3^University of Évora – Nursing School of Évora, Évora, Portugal; ^4^University of Coimbra, Coimbra, Portugal; ^5^Health Sciences Research Unit: Nursing (UICISA:E) Nursing School of Coimbra, Coimbra, Portugal

##### **Correspondence:** Ana Ramos, César Fonseca (cfonseca@uevora.pt)

**Background:** Population aging is a challenge in all the world, in order to better respond to the needs of older people, especially at the level of long-term health care, and new models of services and allocation of resources are needed.

**Objective:** To determine the socio-demographic profile of people aged 65 and over, that used the Convalescence Units, Medium-Duration and Rehabilitation Units, Maintenance and Long-term Units of the Integrated Continuum National Care Network in Portugal

**Method:** A retrospective cross-sectional study, between 2010-2015, using the Statistical Package for Social Sciences (SPSS®) software, version 25.

**Discussion:** From the analysis of the total of 12006 elderly people, it is possible to observe that people over 75 are mostly female. The highest level of education occurs in males; the highest number of people who do not know reading and writing (illiterate) are female and who have the lowest level of education. In terms of age and sex, people who cannot read and write are people over 75 years of age. In both sexes, the social isolation also increases from the 75 years, being more predominant in the women.

**Conclusion:** Health needs are conditioned by multiple variables, such as biological (age, sex) and sociodemographic (education, social support), which will help in later studies to determine its influence on the dependency / independence profile in self care.

**Key words:** People aged 65 and over, sociodemographic profile, long-term care.

## A61 Respiratory rehabilitation gains in persons with respiratory insufficiency submitted to noninvasive ventilation: Systematic review of literature

### Cheila Reis^1^, César Fonseca^1^, Manuel Lopes^1^, Enrique Moguel^2^, Felismina Mendes^1^, Juan M. Murillo^2^, Ana Ramos^3^

#### ^1^University of Évora – Nursing School of Évora, Évora, Portugal; ^2^University of Extremadura, Extremadura, Spain; ^3^Nursing School of Lisbon, University of Lisbon, Lisbon, Portugal

##### **Correspondence:** Cheila Reis, César Fonseca (cfonseca@uevora.pt)

**Background:** Respiratory insufficiency as a condition associated with respiratory disease, whether acute or chronic, conditions and decreases person’s quality of life. Noninvasive ventilation therapy (NIV) plays a key role in the stabilization of respiratory disease, which translates into evident gains for the patient. These will be greater when associated with respiratory rehabilitation therapy. The nurse specialist in rehabilitation, for its specific competencies, plays a fundamental role in the acceptance, effectiveness and adaptation of the patient to NIV.

**Objective:** To describe the gains from the application of associated respiratory rehabilitation of NIV in patients with respiratory failure and the role of rehabilitation nursing.

**Methods:** Seven articles from the scientific databases present on the EBSCO platform, with Full Text, MEDLINE with Full Text and B-on are collected (articles in the last 10 years).

**Results / Discussion:** The importance of NIV in the stabilization of respiratory disease is consensual. It has also been reported in several studies that the use of NIV during exercise can improve tolerance to NIV, however, such information needs further investigation. Respiratory rehabilitation is essential for improving respiratory functionality, and intervention should be performed during and after crisis, with post-discharge follow-up and education to the person and family.

**Conclusion:** NIV is a therapy with recognized advantages in the control of respiratory failure and in the reduction of associated comorbidities. It is safe, effective, comfortable for the patient and applicable to a wide range of acute events and chronic respiratory conditions. Respiratory rehabilitation reduces symptoms, improves respiratory function and consequently person’s quality of life. The nurse specialist in rehabilitation has an important role in the education / information of the person, family and peers.

**Key words:** respiratory rehabilitation, noninvasive ventilation, exercise, tolerance, nurse.

## A63 Nurse staffing in the promotion of the process and quality of healthcare: Nurse managers’ opinion

### Teresa Neves^1^, Pedro Parreira^2^, Victor Rodrigues^3^, Lisete Mónico^3^, Jose García-Alonso^4^, Rogério Ferrinho^5^, João Graveto^2^

#### ^1^Centro Hospitalar e Universitário de Coimbra, Coimbra, Portugal; ^2^Health Sciencies Research Unit:Nursing (UICISA:E) Nursing School of Coimbra, Coimbra, Portugal; ^3^University of Coimbra, Coimbra, Portugal; ^4^University of Extremadura, Extremadura, Spain; ^5^Polytechnic Institute of Beja, Beja, Portugal

##### **Correspondence:** Teresa Neves (te.aneves@gmail.com)

**Introduction**: Nurse staffing influences the process and results of healthcare, with impact in the quality of care. However, little is known about this association in the Portuguese context.

**Objective**: Assess the effect of nurse staffing on the quality of care, mediated by care process.

**Method**: A cross-sectional study was conducted across 71 units in 12 Portuguese hospitals. Nurse managers perception was assessed regarding the adequacy of the nurse staffing (number and competencies), nurses’ response capacity, involvement in continuous improvement projects, patient-centered care process and quality of nursing care. Data were collected from January to September 2015. A path model was used to study the potential causal and mediation, considering the hypotheses: H_1_. nurse staffing (number and competencies) is indirectly associated with quality of care, trough process dimensions; H_2_. Nurses’ response capacity plays a mediating role between nurse staffing and involvement in continuous improvement projects; H_3_. Patient-centered care process has a mediating effect on the relation between the competencies and the involvement in continuous improvement projects, and the quality of care.

**Results:** The model fit has good (χ^2^/df=0.49; CFI=1.00, GFI=0.97, RMSEA=0.00) and explains 53% of the variability in the quality of healthcare, using a sample of 48 nurse managers. Path analysis showed that nursing staff influences process dimensions and, indirectly the quality of care, supporting all the hypotheses. Nurse staffing (number) increased nurses’ response capacity (β=0.459), and indirectly the quality of care (β_total_effect_=0.063). Similarly, competencies increased response capacity (β=0.335), and also had a direct effect on patient-centered care (β=0.344), and an indirect effect on quality of care (β_total_effect_=0.295). Response capacity had a direct pathway in involvement in continuous improvement projects (β=0.470) and an indirect effect on the quality of care (β_total_effect_=0.137). Concomitantly, involvement in improvement projects increased the focus on the patient-centered care (β=0.470), and indirectly promoted quality of care (β_total_effect_=0.292). In addition, patient‑centered care process had a mediating effect on the quality of care (β=0.725).

**Conclusion:** The results reinforce the importance of the nursing staff and the care process to promote the quality of healthcare. This should be considered in health policies and nursing resource management planning.

**Keywords:** nursing staff, hospital, nursing care, patient-centered care, quality of care.

## A64 The influence of emotional intelligence as a factor of protection in Stress: What specificities in healthcare professionals?

### Catarina Cruz^1^, Lisete Mónico^2^, Manuel Lopes^3^, Felismina Mendes^3^, Jose García-Alonso^4^, Henrique Oliveira^5^, Pedro Parreira^1^

#### ^1^Health Sciences Research Unit: Nursing (UICISA:E) Nursing School of Coimbra, Coimbra, Portugal; ^2^University of Coimbra, Coimbra, Portugal; ^3^University of Évora – Nursing School of Évora, Évora, Portugal; ^4^University of Extremadura, Extremadura, Spain; ^5^Polytechnic Institute of Beja, Beja, Portugal

##### **Correspondence:** Catarina Cruz (catarinacruz_821@hotmail.com)

**Introduction:** The globalization of the economy, reductions and adjustments in the organizational structures, leading to challenges, particularly in health services. In this context, the empirical studies indicate a positive impact of emotional intelligence (EI) on people management, with highest expression in healthcare services.

**Objective:** This research exploring the relationship between EI and the perceived stress (PS) in workers, particularly in healthcare professionals.

**Method:** This is an empirical, cross-sectional and non-experimental study, with a non-probabilistic sample by networks of 874 workers from different professional activities (154 healthcare area). The applied questionnaire includes four scales, however in this study we used the EI and PS scales.

**Results:** The scales used with good indexes of adjustment: EI [χ2/df= 3,965; NFI= .923; RMSEA= .055; α= .84; M= 5.11 (SD = .63)]; PS [χ2/df= 4.045; NFI = .953; RMSEA = .059; α = .82; M = 1.79 (SD = .51)]. The results support empirically a negative relationship between emotional intelligence and perceived stress in workers. The relationship between the overall EI and PS Scale is statistically significant and negative in both the total sample (r = -.41) and in health professionals (r = -.49) and also in the other professional categories (r = -.40). With the test of magnitude difference of the correlation coefficients between the EI and PS, we verified the existence of significantly higher negatives in the health professionals [r = -. 49; R^2^ = 24%; p <.01] compared to other health professionals [r = - .40; R^2^ = 16%; p <.01].

**Conclusion:** We conclude that EI has a negative and statistically significant relationship with PS, especially in healthcare professionals. The results obtained suggest a greater awareness among managers and leaders of the need to implement organizational actions that promote the development of EI.

**Keywords:** Emotional intelligence, perceived stress, people management.

## A65 The effects of nurse staffing and work environment on patient safety

### Teresa Neves^1^, Pedro Parreira^2^, Manuel Lopes^3^, Jose García-Alonso^4^, Anabela Salgueiro-Oliveira^2^, Lisete Mónico^5^, João Graveto^2^, Victor Rodrigues^5^

#### ^1^Centro Hospitalar e Universitário de Coimbra, Coimbra, Portugal; ^2^Health Sciences Research Unit: Nursing (UICISA:E) Nursing School of Coimbra, Coimbra, Portugal; ^3^University of Évora-Nursing School of Évora, Évora, Portugal; ^4^University of Extremadura, Extremadura, Spain; ^5^University of Coimbra, Coimbra, Portugal

##### **Correspondence:** Teresa Neves (te.aneves@gmail.com)

**Introduction**: Nurse staffing and practice environment influences the process and results of healthcare, with impact in the patient outcomes, such us patient safety. Scientific evidence identifies the adverse events as an indicator of the safety of care.

**Objective**: Assess the effects of nurse staffing and work environment in adverse events, mediated by nursing practices.

**Method***:* A cross-sectional study was developed across 71 units in 12 Portuguese hospitals. Nurses perception were assessed on nurse staffing adequacy, nurse practice environment and adverse events associated to nursing practices. The data were collected from January to September 2015. A structural equation modelling estimation, using a sample of 793 nurses, was performed to analyze the hypotheses: H_1_. nurse staffing adequacy are negatively associated with risk and occurrence of adverse events both directly and indirectly, trough nursing practice environment; H_2_. nursing practice environment are positively associated with nurses’ practice and negatively associated with risk and occurrence of adverse events; H_3_. nurses’ practice plays a mediating role in the relation between nursing practice environment and risk and occurrence of adverse events.

**Results:** The model showed acceptable overall fit (χ^2^/df=2.94; CFI=0.86, PCFI=0.81; PGFI=0.76, RMSEA=0.05; RNFI=1) and support all hypothesis. 53% of the variance of risk and occurrence of adverse events was explained by the dimensions of the model. The adequacy of nurse staffing was a negatively effect on risk and occurrence of adverse events, directly (β=-0.09), and indirectly (β_total_effect_=‑0.18) through the nurses practice environment (β=-0.26). Work environment was directly associated with nurses’ practice (β=0.29). Moreover, had also influenced directly (β=‑0.19) and indirectly (β_total_effect_=-0,37) the adverse events. The nurses practice seems to play a mediating role between work environment and the risk and occurrence of adverse events (β=‑0.64). All pathways were statistically significant (*p*<.05). Based on the bootstrap resampling method, all the effects were significant (*p*<.05).

**Conclusions**: These findings suggest that optimisation of nurse staffing and nursing practice environment promote the reduction of risk and occurrence of adverse events and the improvement of patient safety. The nursing practices seems to play a mediating role in this relationship. Our findings serve as a support to policies in nursing management.

**Keywords:** nursing staff, hospital; patient safety; work environment; nursing care.

## A66 Gains sensitive to rehabilitation nursing care in dependent person in self-care with comorbidity

### António Lista^1^, César Fonseca^1^, Enrique Moguel^2^, Juan M. Murillo^2^, Pedro Parreira^3^, Felismina Mendes^1^, Manuel Lopes^1^

#### ^1^University of Évora – Nursing School of Évora, Évora, Portugal; ^2^University of Extremadura, Extremadura, Spain; ^3^Health Sciences Research Unit: Nursing (UICISA:E) Nursing School of Coimbra, Coimbra, Portugal

##### **Correspondence:** António Lista, Manuel Lopes (mjl@uevora.pt)

**Background:** Rehabilitation Nursing assumes a fundamental role in contemporary society in the response to the installation of new paradigms of chronic illness and dependence, induced by demographic aging and the advent of a large number of associated comorbidities

**Objective:** To develop acquisition of Rehabilitation Nursing competences and master is competences.

**Method**: A professional intervention strategy was implemented with the purpose of evaluating the sensible gains to the Rehabilitation Nursing care in dependent people with comorbidity.

**Results:** The interventions in both practical contexts were statistically significant, leading to an improvement in the general scores of functionality, providing health gains.

**Conclusions:** The Rehabilitation Nursing competences and master’s competences, initially proposed, are considered to be acquired globally. The strategy implemented promotes professional excellence and is understood as a valid contribution to the theoretical development of Rehabilitation Nursing.

**Keywords**: Rehabilitation Nursing, Competences, Self-Care, Dependency, Health Gains.

## A67 Training for self-care of the elderly in RNCCI: Contributions of rehabilitation nursing care

### Anabela Batista^1^, Felismina Mendes^1^, Manuel Lopes^1^, Jose García-Alonso^2^, Juan M. Murillo^2^, César Fonseca^1^, Ana Ramos^3^

#### ^1^University of Évora – Nursing School of Évora, Évora, Portugal; ^2^University of Extremadura, Extremadura, Spain; ^3^Nursing School of Lisbon, University of Lisbon, Lisbon, Portugal

##### **Correspondence:** Anabela Batista, Manuel Lopes (mjl@uevora.pt)

**Background:** Dependence on self-care commonly related to chronic conditions and aging, are causes of difficulties for users and families, particularly at home. Continuing care in health is one of the challenges present in contemporary societies. The quality of care is focused on one of the concerns that, in addition to clinical efficacy and patient safety, leads to a central care. The RNCCI is a new organizational standard that results in an integrated health response, assuming rehabilitation, rehabilitation and reintegration as intervention objectives.

**Objective**: To present the process underlying the acquisition and development of Rehabilitation and Master's Nursing competences.

**Method**: A professional intervention strategy was implemented in order to evaluate the gains gained in the care of Rehabilitation Nursing, in the training for the self-care of the elderly in the RNCCI.

**Results**: It is certified that through the collected data the interventions of the rehabilitation nurse cooperated to improve the person's functionality, reducing dependence on self-care, providing health gains. Conclusion: It was observed that the competences of Rehabilitation Nursing and Master, initially proposed were fully acquired, contributing to a functional improvement of the people included in the professional intervention strategy.

**Key words**: Self-Care, Elderly, RNCCI, Rehabilitation Nursing

## A68 Earnings from rehabilitation nursing care in people in intensive care with respiratory disorders, based on a Self-Care model

### Marco Jacinto^1^, César Fonseca^1^, Manuel Lopes^1^, Jose García-Alonso^2^, Juan M. Murillo^2^, Felismina Mendes^1^

#### ^1^University of Extremadura, Extremadura, Spain; ^2^University of Évora – Nursing School of Évora, Évora, Portugal

##### **Correspondence:** Marco Jacinto, Manuel Lopes (mjl@uevora.pt)

**Background:** The evolution Portuguese population needs, associated with aging, the increase in the average life expectancy and the appearance of serious diseases, has increased the demand for health care, presenting a greater need for intensive care. The severity of the person's health situation associated with the administration of vasopressors, sedative and muscle relaxants medication are considered as the main precipitating factors of prolonged bed rest, as well as the use of invasive mechanical ventilation. The intervention of the Rehabilitation Nurse encompasses the care provided to the person in critical situation, with the aim of avoiding respiratory, motor and functional complications, thus being central to their intervention, promoting the reconstruction of the autonomy of the people.

**Objectives:** To identify the gains of interventions of rehabilitation nursing care based on the self-care model.

**Method:** Descriptive and exploratory study. Based on the theoretical and methodological assumptions of the case study of Robert Yin (2003), in association with the medium-range theory of Manuel Lopes (2006) and Dorothea Orem's theory of self-care deficit (2001).Target Population: Non-probabilistic sample, consisting of 7 individuals who were provided rehabilitation care by rehabilitation nurses. Data Harvest Instrument: Used the Elderly Core Set (ENCS) and the Barthel Index.

**Results:** It was verified through data analysis that the interventions of the rehabilitation nurse con-tributed to a functional improvement of the person, reducing dependence on self-care.

**Conclusion:** All participants benefited from the intervention of the rehabilitation nurse, improving not only their functionality in general, but also muscle strength and respiratory function.

**Key-words:** Self-Care; Functionality; Rehabilitation Nurse.

## A69 Contribution of rehabilitation nursing care in the improvement of self-care in the hospitalized elderly person

### Christian Krusch^1^, César Fonseca^1^, Jose García-Alonso^2^, Manuel Lopes^1^, Lorenzo Mariano^2^, Pedro Parreira^3^, Felismina Mendes^1^

#### ^1^Nursing School of University of Évora, Évora, Portugal; ^2^Department of Computer Systems and Telematics Engineering, University of Extremadura, Extremadura, Spain; ^3^Health Sciences Research Unit: Nursing (UICISA:E) Nursing School of Coimbra, Coimbra, Portugal

##### **Correspondence:** Christian Krusch, Manuel Lopes (mjl@uevora.pt)

**Background:** Within the scope of the Curricular Unit Report of the Master’s Degree in Rehabilitation Nursing, the report “Contribution of rehabilitation nursing care to improving self-care in hospitalized elderly persons”, this was developed during the Final Stage.

**Methods:** A methodology of care was applied, framed in the medium-range theory of Lopes (2006) and in the model methodology of case study of Robert Yin (2003).

We **objective** to achieve Master's competencies, the Common Nurse Specialist, the specific Nurse Specialist in Rehabilitation Nursing and the provision of specialized nursing care rehabilitation in people with self-care deficit in the context of hospitalization and to establish gains in health.

**Results:** We verified gains in the concepts of self-care, learning and motor function, communication and, also, an increase in functional independence.

**Conclusion:** The effectiveness of specific rehabilitation nursing interventions, contributing positively to the improvement of self-care and functionality as well as the state of global dependence on the daily activities of the elderly person.

**Keywords:** Self-care, Rehabilitation Nursing, Aging, Elderly.

## A70 A structured proposal for the intervention of rehabilitation nursing care, for elderly people with a deficit in self-care and motor disorders

### Anabela Batista^1^, César Fonseca^1^, Jose García-Alonso^2^, Lisete Mónico^3^, Juan M. Murillo^2^, Manuel Lopes^1^, Ana Ramos^4^

#### ^1^University of Évora – Nursing School of Évora, Évora, Portugal; ^2^University of Extremadura, Extremadura, Spain; ^3^University of Coimbra, Coimbra, Portugal; ^4^Nursing School of Lisbon, University of Lisbon, Lisbon, Portugal

##### **Correspondence:** Anabela Batista, Manuel Lopes (mjl@uevora.pt)

**Background**: The concept of functional capacity is particularly useful in the context of aging. Aging while maintaining all its functions does not mean a problem for both the individual and the community, when their functions begin to deteriorate, problems begin to emerge. The concept is closely linked to the maintenance of the autonomy from which the potential loss of functionality and the increasing prevalence of chronic diseases, such as motor diseases, can be maintained, thus enabling the person to perform his or her self-care in the sense of independence, a primary goal of Rehabilitation Nursing. The quality of care is one of the targets of Rehabilitation Nursing, confirming its effectiveness when showing results sensitive to Nursing care.

**Objective:** to develop skills in the area of Rehabilitation Nursing care, through structured intervention plans for elderly people with a deficit in self-care and mobility disorders.

**Methods:** The present study is descriptive and exploratory, based on the qualitative methodology of the case study (multiple case study method) by Robert Yin [1] and Lopes’ [2] medium-range theory, based on the theory of self-care deficit of Orem [3].

**Results:** Considering the increase in the consequences of nursing care, such as functionality, self-care and patient satisfaction, in addition to the user's capacity for self-management of chronic illness, through rehabilitation programs, teaching, prevention of return to health services, decreasing the amounts related to the health system.

**Conclusion:** The structured interposition of Rehabilitation Nursing care, based on a program of motor functional reeducation, empowerment of the person, translates into gains in self-care, at the level of motor function.

**Key words:** Sensitive results, Nursing care, Rehabilitation, altered motor function

**References**

1. Yin R. *Case study research: Design and methods* (3 edª). Thousand Oaks: Sage; 2003.

2. Lopes M. *A relação enfermeiro-doente como intervenção terapêutica*. Coimbra: Formasau – Formação e Saúde, LDA.; 2006.

3. Orem D. *Nursing: Concepts of practice*. St. Louis: Mosby; 2001.

## A71 A structured proposal for rehabilitation nursing care intervention for elderly people with self-care deficit and respiratory disorders

### Vânia Dias^1^, César Fonseca^1^, Rogério Ferrinho^2^, Manuel Lopes^1^, Jose García-Alonso^3^, Juan M Rodríguez^3^, Ana Ramos^4^

#### ^1^Nursing School, University of Évora, Évora, Portugal; ^2^Polytechnic Institute of Beja, Beja Portugal; ^3^University of Extremadura, Extremadura, Spain; ^4^Nursing School of Lisbon, University of Lisbon, Lisbon, Portugal

##### **Correspondence:** Vânia Dias, Manuel Lopes (mjl@uevora.pt)

**Introduction**: Due to an aging population, from which stems the potential loss of functionality and the increasing prevalence of chronic diseases, particularly respiratory diseases, empowering people to perform self-care and promoting their independence becomes a primary objective of Rehabilitation Nursing. The quality of care is a major goal of Rehabilitation Nursing and its effectiveness has been proven when showcasing nursing care-sensitive results.

**Objective:** Developing skills in the area of Rehabilitation Nursing care through structured intervention plans, aimed at elderly people with self-care deficits and respiratory disorders.

**Methods:** The present study is both descriptive and exploratory, based on the qualitative methodology case study applied by Robert Yin in his multiple case study method [1], as well as Lopes’ medium-range theory [2] which was based on Orem’s theory of self-care [3].

**Results:** A significant increase in nursing care-sensitive outcomes such as functionality, self-care and patient satisfaction was registered, in addition to enabling the client to self-manage chronic illness through proper education, preventing subsequent recurrences to health services which in turn reduced the overall costs incurred by the national health system.

**Conclusion:** The structured intervention of Rehabilitation Nursing care, based around a respiratory function re-education program targeting both the person and his/her family caregiver should directly translate into gains in terms of self-care, learning and mental functions, along with an overall improvement of respiratory function.

**Keywords:** Sensitive results, nursing care, rehabilitation, changes in respiratory function

**References**

1. Yin R. *Case study research: Design and methods* (3 edª). Thousand Oaks: Sage; 2003.

2. Lopes M. *A relação enfermeiro-doente como intervenção terapêutica*. Coimbra: Formasau – Formação e Saúde, LDA.; 2006.

3. Orem D. *Nursing: Concepts of practice*. St. Louis: Mosby; 2001.

## A72 Gains to the care of Nursing patients with anti-aging of self-employed in anti-aging and surgical process: Structured intervention proposal

### Vânia Nascimento^1^, Manuel Lopes^1^, César Fonseca^1^, Felismina Mendes^1^, Jose García-Alonso^2^, Juan M. Murillo^2^, Ana Ramos^3^

#### ^1^Nursing School of Évora, University of Évora, Évora, Portugal; ^2^University of Extremadura, Extremadura, Spain; ^3^Nursing School of Lisbon, University of Lisbon, Lisbon, Portugal

##### **Correspondence:** Vânia Nascimento, Manuel Lopes (mjl@uevora.pt)

**Background:** Surgical needs have been increasing worldwide due to population aging, chronic diseases increase and also by the evolution of knowledge and technology in the surgical area, showing a decrease in the functionality of people in the surgical process and consequent increase in dependence on self-care. The intervention of rehabilitation nursing has as its primary objective the empowerment of the person to perform their self-care, making it imperative to demonstrate the effectiveness of this intervention through the demonstration of the results sensitive to Rehabilitation Nursing care, proving the quality of care provided.

**Objective:** to develop skills in the area of ​​rehabilitation nursing care and to evaluate the sensitive gains to rehabilitation nursing care in persons with a deficit in self-care and in the surgical process, through the elaboration and implementation of a structured proposal of intervention of the nursing care of rehabilitation.

**Methods:** The present study is descriptive and exploratory, based on the qualitative methodology of the case study (multiple case study method) by Robert Yin and Lopes ‘theory of medium range, based on Orem’s theory of self - care.

**Results:** Significant increase in nursing care-sensitive outcomes such as functionality, self-care, and the satisfaction of the person with the rehabilitation nursing care provided, in addition to training the person through teaching, preventing further recurrences to health services, decreasing costs in the health system.

**Conclusion:** The structured intervention of rehabilitation nursing care, based on a program of functional re-education and empowerment of the person and their caregiver / family, translates into gains in functionality, self-care, learning and mental functions, friends and caregivers and in communication.

**Keywords:** Self-care, Rehabilitation nursing, Health gains.

## A73 Virtual technology: Teaching and learning strategy

### Cláudia M Messias, Carolina L Santos, Bianca D Vieira, Ana K Brum, Daniele M Silva

#### Fluminense Federal University, Rio de Janeiro, Brazil

##### **Correspondence:** Cláudia M Messias ( marimessi1512@gmail.com)

**Introduction:** Practical theoretical teaching is a training phase that develops skillsand abilities for nursing professionals.

**Objective:** to develop a website as a tool to assist in the theoretical-practical teaching of the discipline of women's health and to evaluate their efficiency in the construction of knowledge.

**Methods:** Monitoring activity that occurred with the creation of a website, at the link https://focoemmulher2.wixsite.com/meusite, containing information relevant to the students for the practice of the discipline of women’s health, assisting both in theory and in practice. After a concise search of books and manuals in the area, abstracts and textbooks focused mainly on the three central characters of the women’s health area II were produced: pregnant women, puerperal women and newborns, without disregarding relevant information female universe, such as the climacteric, violence against women and breast cancer / uterus.

**Results:** The website had the main interfaces: pregnant, puerperal and newborn. In the category “pregnant” there is a subdivision in physical examination; guidelines; non-invasive technologies and physical examination example. The category “puerpera” is subdivided into care; physical exam; guidelines and example of evolution. Finally, in the “newborn” category, abstracts are geared toward physical examination and guidelines. The evaluation of the website was based on the virtual form, which contained the following items: assessment of the didactics of presentation of content on the website; content development through the website; numerical evaluation of the student to the website, with a score of 0to 10 and personal suggestions and criticisms. Twenty-three students participated in the evaluation process. 100% evaluated that the information on the website is clear and objective; 100% confirmed that the site development was adequate, ensuring ease of access. 70% evaluated the site with a grade of 10(ten), 20% with a grade of 9 (nine) and 10% with a grade of 8.

**Conclusion:** the project made possible the development of a virtual technology that proved to facilitate the teaching and learning process, work of the nursing monitor.

## A74 Henrique Autran and advertising strategies of the National Service for Health Education in the decade of 1920

### Juliana Gama^1^, Renato da Silva^1^, Lucia Lourenço^2^, Maria Gomes^2^

#### ^1^University of Grande Rio – UNIGRANRIO, Rio de Janeiro, Brazil; ^2^Faculty of Bezerra de Araújo, Rio de Janeiro, Brazil

##### **Correspondence:** Juliana Gama (jupublicitaria@gmail.com)

**Introduction:** The health reform developed between 1920 and 1924 in Brazil was in response to the sanitary chaos installed in 1918. The National Department of Public Health (DNSP) was created with sanitary power and autonomy to implement sanitary public policies. A worldwide movement in developed countries was under way. The state of Bahia was the pioneer to launch sanitary campaigns of good hygiene. Public policies have invested in the dissemination of better habits through advertising strategies. The National Health Education Service (SNES) was created, subordinated to the DNSP, to be responsible for the elaboration and supervision of activities specifically aimed at health education. The physician Henrique Autran, with a journalistic background, came from Bahia to put his knowledge into practice in both areas, and head the SNES. He used the technological means available at the time: the radio and the cinema.

**Objective:** to describe the informative advertisements developed by SNES to modify health education practices in Brazil, coordinated and guided by the hygienist physician Henrique Autran. Method: The sources were articles, dissertations and publications on the history of the creation of the SNES, in the period of the first republic in Brazil, and health campaigns prepared and articulated with the society by the SNES.

**Results:** From the hygiene delegate to the head of the health propaganda and health service, Dr. Henrique Autran unified information vehicles, gave lectures and conferences, distributed newspapers, pamphlets and magazines in one purpose. Advertising and publicity were the strategic means to develop habits of prevention and treatment of diseases.

**Conclusions:** The study made it possible to better understand the advertising strategies developed in the beginning of the 1920s, as well as to understand the choice of Bahian physicians to direct the SNES, who brought in their professional baggage the expertise practiced in their home state. It can be seen after the creation of the SNES, a significant advance in public health policies to strengthen health reform in progress, the monitoring of the evolution of advertising in time, such as advances in the field of health care and public health in Brazil.

## A75 The structural modifications in the asylum building of mendicity for general hospital: 1923

### Juliana Gama^1^, Aline Cal^2^, Lucia Lourenço^3^, Maria Gomes^3^

#### ^1^University of Grande Rio – UNIGRANRIO, Rio de Janeiro, Brazil; ^2^Hospital Pró Cardíaco, Rio de Janeiro, Brazil; ^3^Faculty of Bezerra de Araújo, Rio de Janeiro, Brazil

##### **Correspondence:** Juliana Gama (jupublicitaria@gmail.com)

**Introduction:** The building of the old Asylo da Mendicidade, built in 1879 to house beggars and orphans in 1923, was built in the city of Rio de Janeiro in 1879 and had the necessary physical facilities to serve as nursery field at the Anna Neri School. in the General Hospital.

**Objective:** to identify the structural modifications made in the Asylo building to meet the needs of the General Hospital to attend nurses’ training.

**Method:** The sources were documents belonging to the collections of the Hospital São Francisco de Assis, the Documentation Center of the Anna Nery School and the National Library.

**Results:** In the nineteenth century Rio de Janeiro underwent several structural adjustments to house the Portuguese court. The monoblock building, in Neoclassical style, with two floors, panótico model, with a pavilion with body in the central area of the facade, and two rays with two floors distributed laterally. The building of the Asylo was adequate to function like General Hospital: in the second floor were removed the catwalks that connected the central body of the construction to the rays of the panótico; the songs are different; on the floor of the first floor there are marks of the cast iron pillars that supported the catwalks; two ladders were built connecting the pavements; the deposit of oxygen bullets under the stairs demonstrates use of space and adaptation to the hospital environment; ladders were built in the passageway “diagonally” cutting the lower floor windows; were building annexes with bathrooms; the window and floor masonry are incompatible with the style of the initial construction; were built platibanda with architectural cover of ornamentation not common for season. Conclusions: Eclecticism is recognized by the mixture of materials in the same architectural construction. The structural modifications carried out in the Asylo building were fundamental for the development of modern nursing, for medicine and for health quality in Brazil, which, at that time, required qualified and resolute public health care to alleviate the health demand of the population of Rio de Janeiro. The building was listed by IPHAN 1988, and for twenty years it has been restored.

